# Divergent Responses of Bacterial Communities to Permafrost Degradation and Their Associations With Carbon Across Vertical Profiles

**DOI:** 10.1002/advs.202510516

**Published:** 2026-02-15

**Authors:** Shengyun Chen, Yuzheng Gu, Ali Bahadur, Enyan Liu, Tonghua Wu, Xiaofan Zhu, Yuanqiang Zou, Hewei Liang, Peijie Wei, Linwei Wu, Qingbai Wu, Peizhi Yang, Hongyan Yu, Yunfeng Yang

**Affiliations:** ^1^ Cryosphere and Eco‐Environment Research Station of Shule River Headwaters, State Key Laboratory of Cryospheric Science and Frozen Soil Engineering, Northwest Institute of Eco‐Environment and Resources Chinese Academy of Sciences Lanzhou Gansu China; ^2^ College of Ecology Lanzhou University Lanzhou Gansu China; ^3^ College of Grassland Agriculture Northwest A&F University Yangling Shaanxi China; ^4^ Cryosphere Research Station on the Qinghai‐Tibet Plateau Chinese Academy of Sciences Lanzhou Gansu China; ^5^ State Key Laboratory of Genome and Multi‐omics Technologies BGI Research Shenzhen China; ^6^ Institute of Ecology, Key Laboratory for Earth Surface Processes of the Ministry of Education, College of Urban and Environmental Sciences Peking University Beijing China; ^7^ Long‐term National Scientific Research Base of the Qilian Mountain National Park Xining Qinghai China; ^8^ Institute of Environment and Ecology, Tsinghua Shenzhen International Graduate School Tsinghua University Shenzhen China; ^9^ University of Chinese Academy of Sciences Beijing China

**Keywords:** alpine permafrost degradation, active and permafrost layers, bacterial assembly processes, community stability, carbon storage

## Abstract

Permafrost degradation poses a significant threat to the organic carbon (C) pool primarily through regulating microorganisms. However, microbial responses and their associations with C loss across vertical profiles remain unclear. Here, we use metagenomic sequencing to investigate bacterial communities in 125 samples from five 15 m‐depth permafrost cores, spanning from the active layer to the permafrost layer along a degradation gradient on the Qinghai‐Tibet Plateau. We find that *α*‐diversity decreases, while stochastic processes and community stability increase from the active layer to the permafrost layer. Along permafrost degradation, these community attributes follow similar variations within the active layer but remain constant within the permafrost layer. The relative abundance and interaction of core taxa play important roles in maintaining community stability in the active and permafrost layers, respectively. As permafrost degrades, the negative relationships between community stability and C storage become more intense, especially in the active layer. These findings demonstrate that degradation induces microbial responses that potentially amplify C release, supporting a positive feedback loop to climate warming. Our work provides novel insights into the vertical heterogeneity of this mechanism and is crucial for modeling future permafrost C dynamics.

## Graphical abstract

Bacterial α‐diversity decreases, but stochasticity and community stability increase across the 15 m‐depth vertical profiles and along the degraded gradient within the active layer. The abundance and interaction of core taxa mainly control community stability in the active and permafrost layers, respectively. Community stability shows significant and negative associations with carbon storage, especially in the active layer.

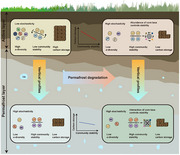

## 1 Introduction

Microbial communities play a crucial role in driving essential ecological processes and functions in terrestrial ecosystems, such as organic carbon (C) turnover, primary productivity, and nutrient cycling [1, 2, 3, 4]. As a result, the functioning and sustainability of soil ecosystems are critically influenced by microbial community stability, which represents the degree of variation or turnover rate within these communities [5]. Accumulating evidence suggests that community stability is primarily attributed to species diversity, network properties, and assembly processes [6, 7, 8, 9]. Network properties (e.g., complexity, centrality, and modularity) characterize possible ecological interactions among community members [10, 11]. Community assembly refers to the processes by which species from a regional pool colonize and interact to establish and maintain local communities. It typically encompasses deterministic processes (e.g., environmental selection driven by abiotic factors like pH, temperature, or biotic interactions such as competition and mutualism) and stochastic processes (e.g., ecological drift, dispersal limitation, and random birth/death events) [12, 13]. These community attributes have been extensively applied in the field of microbial ecology [14, 15]. Although it is widely accepted that more diverse and interactively complex communities with higher deterministic processes tend to be more stable [5, 7, 16], recent research has presented conflicting evidence that reduced diversity, increased stochastic processes, and simplification of interaction networks can lead to an enhancement of community stability [17, 18, 19]. This discrepancy may be attributed to the differences in environmental conditions and ecosystem types [19]. Therefore, given the escalating challenges posed by climate change, such as accelerated warming rates and altered precipitation patterns, elucidating microbial dynamics is especially critical in vulnerable environments such as permafrost, which acts as a sensitive indicator of climate change [20, 21].

Permafrost, a major component of Earth's terrestrial cryosphere, is defined as ground (soil or rock) that remains at or below 0°C for at least two consecutive years [22], serving as a unique habitat for microbial life compared with other ecosystems [23]. However, research on permafrost microbial communities has mainly concentrated in high‐latitude regions (e.g., Alaskan and Canadian Arctic), neglecting the distinctiveness of permafrost in high‐altitude regions [24, 25]. The Qinghai‐Tibet Plateau (QTP) possesses the world's largest areas of middle‐low latitude and high‐altitude mountain (alpine) permafrost [26], characterized by a thicker active layer (212.03 cm in the QTP and 100.18 cm in the Arctic) and a higher mean annual ground temperature (MAGT) (‐1.9°C in the QTP and −5.9°C in the Arctic) compared to its high‐latitude counterparts [27]. These unique conditions are highly likely to yield distinct microbial patterns. Furthermore, our current understanding of microbial community attributes in the QTP is still focused on the active layer [9, 24], leaving a significant knowledge gap regarding the permafrost layer, particularly at deeper depths. Specifically, the frequent freeze–thaw cycles and liquid water availability in the active layer facilitate microbial interaction and niche selection, thereby enhancing deterministic processes [28, 29]. In contrast, although the harsh environment of the permafrost layer filters for stress‐tolerant taxa, the consecutively frozen state imposes severe dispersal limitation and physical isolation. These constraints override environmental selection, allowing stochastic processes to dominate community assembly in the deeper permafrost layer [30, 31]. Importantly, the frozen fringe layer or transition layer, positioned between the unfrozen and frozen zones, exhibits high sensitivity to climate change because it serves as the dynamic thermal interface where phase changes occur [32, 33]. As the active layer thickens due to warming, this interface migrates downward, regulating the critical exchange of water and heat between the thawing soil and the underlying permafrost [32, 33]. However, the frozen fringe layer has received little attention. These gaps arise from the challenges involved in sampling deep permafrost, and extracting DNA from samples with low biomass [23]. Hence, despite a longstanding interest in permafrost microorganisms, their patterns across vertical profiles in alpine permafrost remain unresolved.

Permafrost degradation is becoming increasingly prevalent across the QTP due to climate warming, as evidenced by the rise in MAGT, thawing of the permafrost layer, and thickening of the active layer [27, 34]. This trend severely threatens the huge C pool in permafrost, which contains 14.1 Pg organic C down to 3 m‐depth in the QTP region, of which 12.3 and 1.8 Pg organic C are stored in the active layer and permafrost layer, respectively [35]. Importantly, permafrost degradation enhances microbial‐mediated decomposition of organic matter, increasing greenhouse gas emissions. This amplifies climate warming, which in turn accelerates further permafrost degradation, triggering a positive feedback [25, 36]. Previous research has revealed that permafrost degradation considerably reduced microbial diversity and stability in the surface of the active layer, which were significantly associated with C loss [9]. Another recent study also has demonstrated that deterministic processes are closely linked to the greenhouse gas emissions over a permafrost thaw gradient, highlighting the role of microbial ecological processes in regulating permafrost C dynamics [29]. However, substantial uncertainties persist concerning the applicability of these observed patterns in the active layer to the permafrost layer [25]. By analogy with the evidence that aridity could impact deep microbial communities and their associated ecological functions [37], we propose that these variations induced by permafrost degradation at the surface would also propagate into the deeper permafrost layer. Additionally, some studies have found that core taxa, typically defined as the microbial taxa that are both highly abundant and consistently present across the majority of samples within a given habitat, can affect ecological functions by increasing community stability [38, 39]. Whether core taxa enhance community stability to mediate C cycling in permafrost has not been explored. Thus, there is a lack of comprehensive information on the relationship between communities and C storage across vertical profiles of permafrost amid degradation.

**FIGURE 1:**
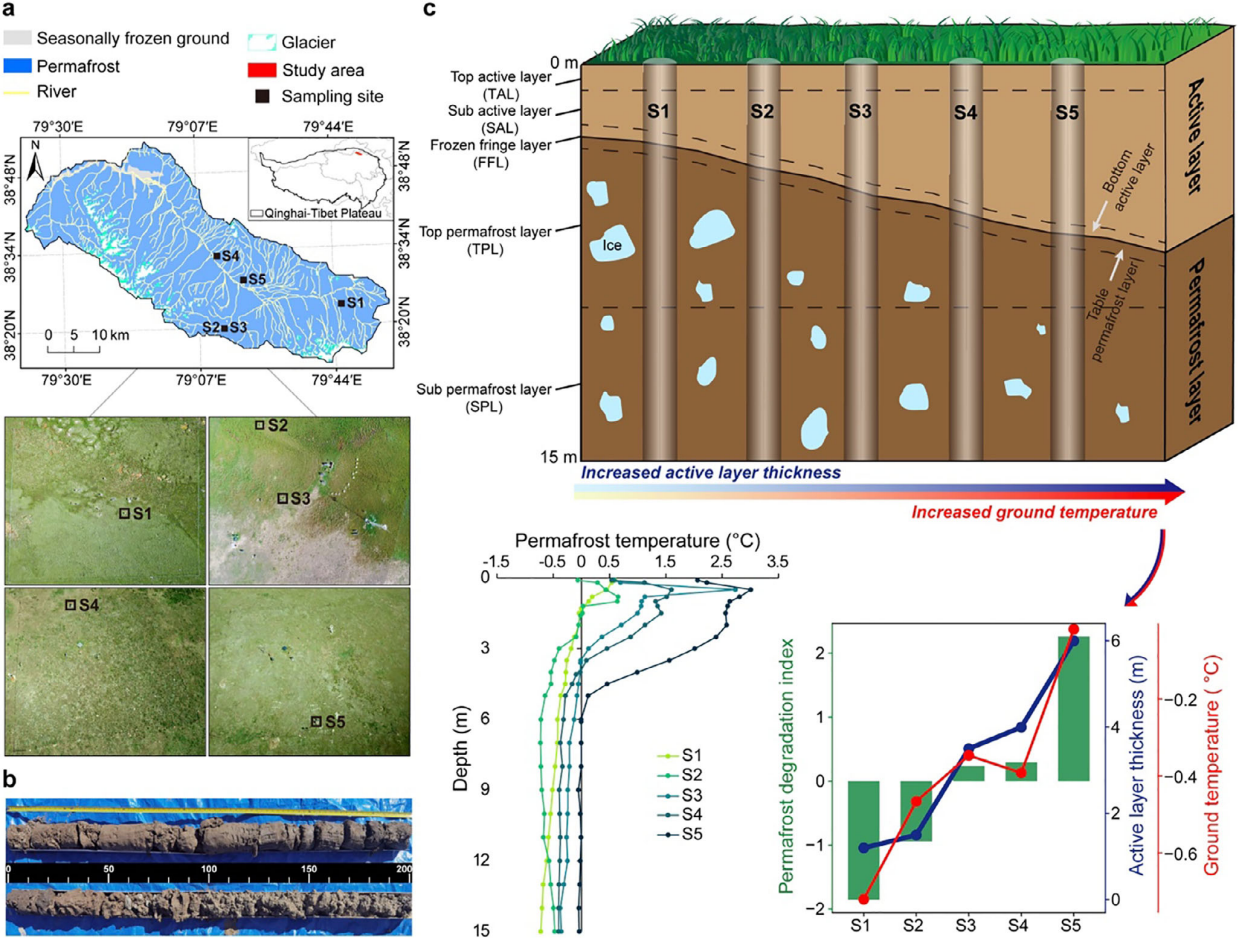
Schematic map of the permafrost sampling. (a) Spatial distribution of sampling sites. (b) Permafrost cores retrieved by a vibratory coring device. (c) Conceptual figures showing the base features of permafrost profiles, the degradation degrees of the five sampling sites, and the grouped information of collected samples. Permafrost degradation index is the first component obtained from the active layer thickness and average mean ground temperature at 15 m depth using principal component analysis and is used to rank the degrees of permafrost degradation of the five sampling sites, in ascending order from S1 to S5 to represent the degradation gradient. The permafrost profiles are divided into two main‐layers including the active layer and the permafrost layer, and are further categorized into five sub‐layers, including top‐active layer, sub‐active layer, the frozen fringe layer, top‐permafrost layer, and sub‐permafrost layer.

To advance our understanding of alpine permafrost microorganisms and their ecological effects, we investigated bacterial communities in five 15 m‐depth permafrost cores, encompassing the active and permafrost layers. Sampling was carried out during the fall in the Shule River headwaters, on the western part of the Qilian Mountains, northeast margin of the QTP, China (Figure 1a–c and Table S1). To take a closer examination of the community variations across vertical profiles, we divided the profiles into five distinct sub‐layers, including the top‐active layer, sub‐active layer, frozen fringe layer, top‐permafrost layer, and sub‐permafrost layer. Based on the permafrost degradation index (PDI, calculated by active layer thickness and MAGT together), the five sampling sites were ordered from S1 to S5, representing a degradation gradient (Figure 1c). We aimed to test three main hypotheses: (1) Bacterial *α*‐diversity would decrease, but stochastic processes and community stability would increase from the active layer to the permafrost layer. (2) These community attributes within the active layer would show higher sensitivity to permafrost degradation compared with the permafrost layer. (3) The relationship between communities and C storage would be more robust in the active layer than the permafrost layer, as well as along the degradation gradient.

## 2 Results

### 2.1 Permafrost Properties

Permafrost properties exhibited substantial variations across vertical profiles extending to 15 m depths, with permafrost temperature (Tp), permafrost water content (PWC), permafrost organic matter (POM), and total nitrogen (TN) being significantly greater in the active layer than in the permafrost layer. Conversely, pH was significantly lower in the active layer than in the permafrost layer, while redox potential (Eh) showed no significant differences (Figure S1a). Moreover, PWC, POM, and TN displayed dramatic variations from the top‐active layer to the sub‐active layer, with less variability observed from the frozen fringe layer to the sub‐permafrost layer (Figure S1a). Along the degradation gradient based on the increased PDI from S1 to S5, there was a significant increase in Tp but decreases in PWC, POM, and TN within the active layer (Figure S1b). Within the permafrost layer, Tp and PWC increased significantly with degradation (Figure S1c).

### 2.2 Bacterial Community Structure and Assembly Processes

We found that the major phyla of bacterial communities were consistent across five permafrost cores down to 15 m depths. Except for Firmicutes, the relative abundances of these phyla exhibited significant variations across the main‐layers (from the active layer to the permafrost layer) and the sub‐layers (from the top‐active layer to the sub‐permafrost layer) (Figures S2 and S3a, and Table S2). For instance, the relative abundances of Proteobacteria and Actinobacteria exhibited a substantial increase (from 52.4% to 73.6%) and a decline (from 16.6% to 2.9%) from the top‐active layer to the bottom‐permafrost layer, respectively (Figure S3a). Nevertheless, only the relative abundance of Actinobacteria within the permafrost layer displayed pronounced alterations along permafrost degradation (Figure S3b,c and Table S2). We also performed the linear trend analysis to evaluate the bacterial variations at the species level. Our results showed that the relative abundances of *Acinetobacter sp. MYb10* and *Ralstonia solanacearum* significantly increased from the top‐active layer to the sub‐permafrost layer, while those of *Terriglobus roseus* and *Paludibaculum fermentans* exhibited the opposite changes (Figure S4a). In addition, we observed a significant decline in the relative abundances of *Geobacter pickeringii* and *Desulfomicrobium baculatum*, but an increase in those of *Rubrobacter sp. SCSIO_52909* and *Sphingomonas melonis* as permafrost degraded within the active layer (Figure S4b). Even within the permafrost layer, many species showed significant changes along the degradation gradient, such as *Hymenobacter qilianensis* and *Nitrospira moscoviensis* (Figure S4c).

**FIGURE 2:**
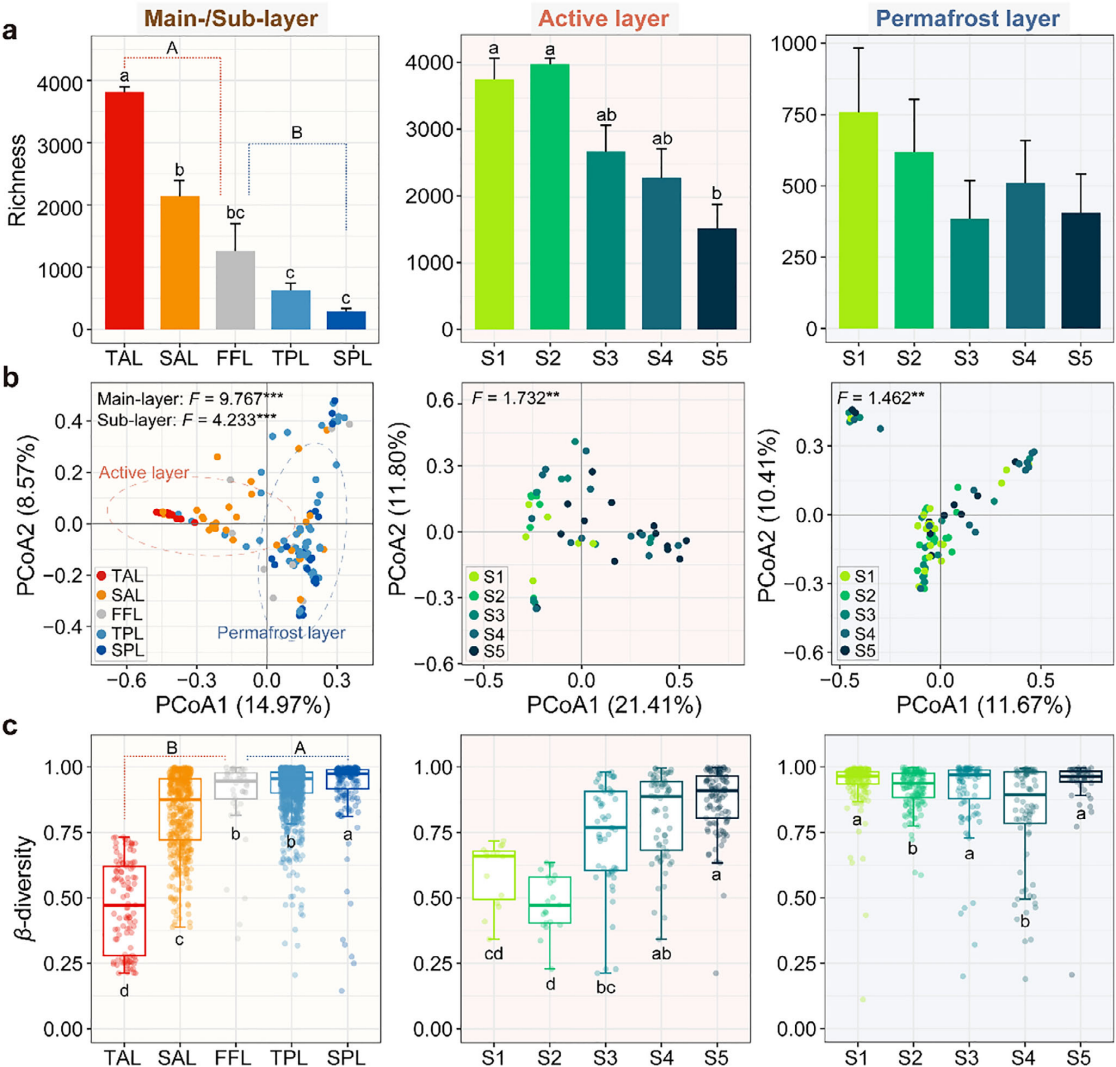
*α*‐ and *β*‐diversity of bacterial communities. (a) Variations in bacterial richness across the main‐ and sub‐layers, and the gradient of permafrost degradation. (b) Variations in bacterial composition based on the Bray‐Curtis distance across the main‐ and sub‐layers, and the degradation gradient shown in PCoA plots and tested by PERMANOVA. In the left panel, PERMANOVA tests were performed based on main‐layers (active and permafrost layers) and sub‐layers (TAL, SAL, FFL, TPL, and SPL). In the middle and right panels, PERMANOVA tests were performed based on sites (from S1 to S5) within the active and permafrost layers, respectively. Asterisks indicate statistical significance of PERMANOVA tests (***p < 0.001, **p < 0.01, and *p < 0.05). (c) Variations in *β*‐diversity across the main‐ and sub‐layers, and the degradation gradient. TAL, top‐active layer; SAL, sub‐active layer; FFL, frozen fringe layer; TPL, top‐permafrost layer; SPL, sub‐permafrost layer. TAL, SAL, and the upper part of FFL (bottom‐active layer) belong to the active layer, the lower part of FFL (table‐permafrost layer), TPL, and SPL belong to the permafrost layer. The sites were ordered from S1 (least degraded) to S5 (most degraded). Sample sizes of sites in the active layer are *n* = 6, 7, 11, 12, and 15 for S1, S2, S3, S4, and S5, respectively. Sample sizes of sites in the permafrost layer are *n* = 19, 18, 14, 13, and 10 for S1, S2, S3, S4, and S5, respectively. Boxplots show median and interquartile range. Data are presented as mean ± s.e.m. Statistical significance is based on Kruskal–Wallis tests; Lowercase letters represent the significance of differences among sub‐layers and among sites in the active and permafrost layers, and uppercase letters represent the significance of differences between main‐layers (active and permafrost layers).

Bacterial *α*‐diversity, including both the richness and Shannon index, was significantly higher in the active layer than the permafrost layer, with a notable reduction from the top‐active layer to the sub‐active layer, but no obvious variations from the frozen fringe layer to the sub‐permafrost layer (Figures 2a and Figure S5a). As permafrost degraded, a decline in *α*‐diversity was observed within the active layer, while the permafrost layer showed no significant change (Figure 2a). Principal coordinate analysis (PCoA) and permutational multivariate analysis of variance (PERMANOVA) revealed significant shifts in community composition across the main‐ and sub‐layers, as well as along the degradation gradient within both the active and permafrost layers (Figure 2b). Specifically, bacterial community dispersions and *β‐*diversity (dissimilarities) markedly increased across the main‐ and sub‐layers, with significant increases within the active layer amid degradation (Figure 2c and Figure S5b). In addition, core bacterial taxa were identified based on occurrence frequency and relative abundance. Core taxa were dominated by Proteobacteria, Bacteroidetes, and Firmicutes, both in terms of taxa number and relative abundance (Figure S6a). At genus level, *Ralstonia* and *Pseudomonas* were the primary constituents (Figure S6b). Detailed taxonomic classifications for each species‐level core taxon were provided in Table S3. We also found that the relative abundance of core taxa significantly increased through the main‐ and sub‐layers, and the degradation gradient (Figure S6c).

**FIGURE 3:**
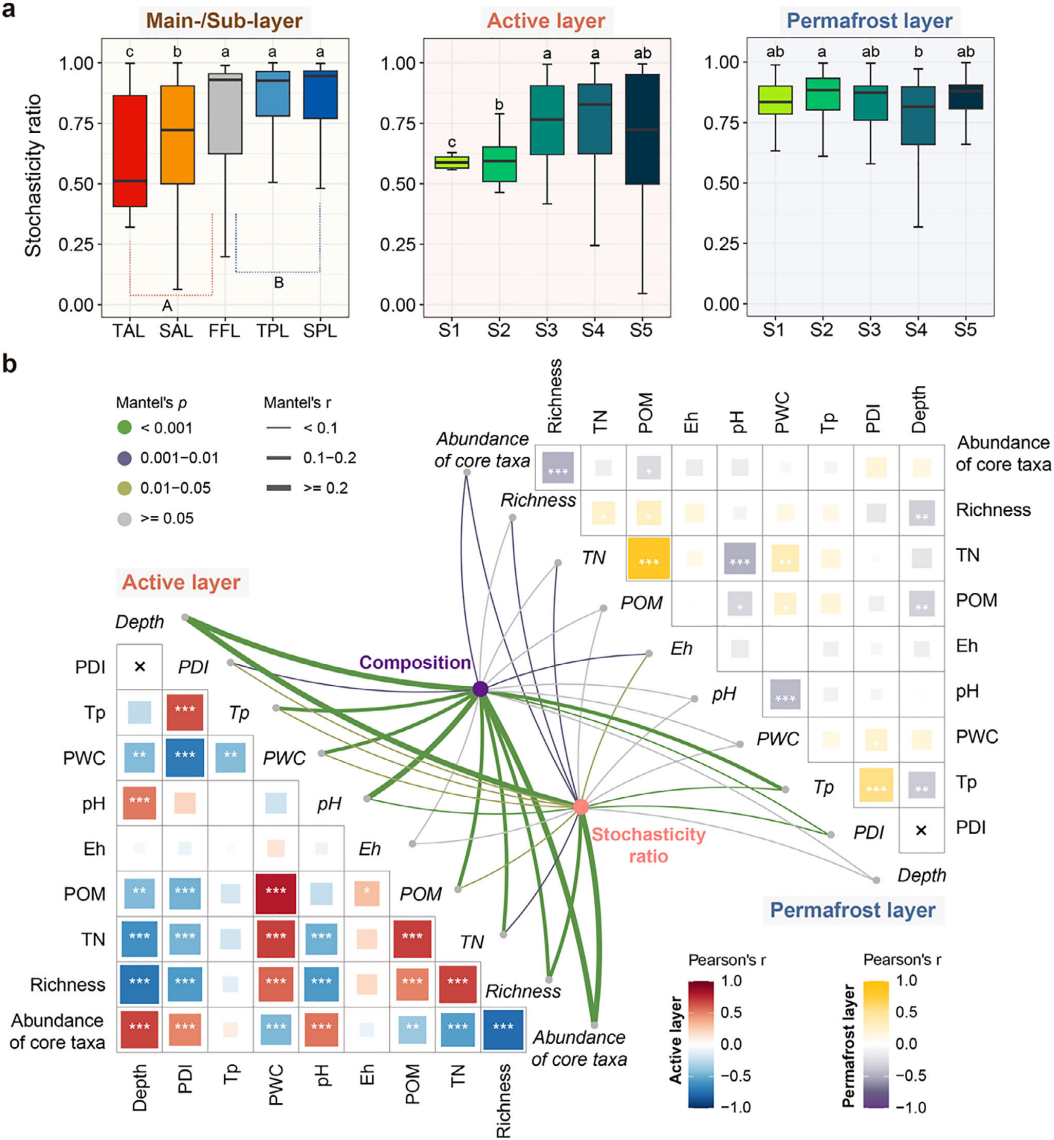
Bacterial assembly processes and the affecting variables of composition and stochasticity ratio. (a) Variations in stochasticity ratio across the main‐ and sub‐layers, and the gradient of permafrost degradation. The stochasticity ratio is calculated for each pair of samples based on the Bray ‐–Curtis distance. (b) Correlations of bacterial composition (Bray‐Curtis dissimilarity) and stochasticity ratio with environmental variables, richness, and abundance of core taxa in the active and permafrost layers based on the best models from the modified Mantel analysis (see Table S3 for details). PDI, permafrost degradation index; Tp, permafrost temperature; PWC, permafrost water content; Eh, redox potential; POM, permafrost organic matter; TN, total nitrogen. TAL, top‐active layer; SAL, sub‐active layer; FFL, frozen fringe layer; TPL, top‐permafrost layer; SPL, sub‐permafrost layer. TAL, SAL, and the upper part of FFL (bottom‐active layer) belong to the active layer, the lower part of FFL (table‐permafrost layer), TPL, and SPL belong to the permafrost layer. Sample sizes of sites in the active layer are *n* = 6, 7, 11, 12, and 15 for S1, S2, S3, S4, and S5, respectively. Sample sizes of sites in the permafrost layer are *n* = 19, 18, 14, 13, and 10 for S1, S2, S3, S4, and S5, respectively. The sites were ordered from S1 (least degraded) to S5 (most degraded). Boxplots show median and interquartile range. Data are presented as mean ± s.e.m. Statistical significance is based on Kruskal–Wallis tests; Lowercase letters represent the significance of differences among sub‐layers (TAL, SAL, FFL, TPL, and SPL) and among sites (from S1 to S5) in the active and permafrost layers, and uppercase letters represent the significance of differences between main‐layers (active and permafrost layers). Asterisks indicate statistical significance (***p < 0.001, **p < 0.01, and *p < 0.05).

Bacterial community assembly was primarily governed by stochastic processes, which were prominently higher in the permafrost layer than the active layer (Figure 3a). Specifically, there was a notable escalation in the stochasticity ratio from the top‐active layer to the frozen fringe layer, followed by no significant changes up to the sub‐permafrost layer. Along the gradient of permafrost degradation, the stochasticity ratio showed a substantial increase within the active layer but not within the permafrost layer (Figure 3a). Furthermore, noticeable decreases in migration rate and niche overlap were observed across the main‐ and sub‐layers, and the degradation gradient (Figure S7a,b). These indicated the reduced community dispersal between habitats [40], and shared resource utilization or ecological interactions among microbial members [41], demonstrating a decrease in the deterministic processes. Meanwhile, the changes in community similarities were more pronounced and sharper in the active layer than the permafrost layer over increasing Euclidean distances based on permafrost properties, depth, and PDI, respectively (Figure S8). It suggested that bacterial communities in the active layer were more susceptible to spatial variation and environmental filtering [42, 43], with a more unstable structure [9, 44]. The modified Mantel test revealed significant correlations between bacterial community composition and the stochasticity ratio and all variables except Eh in the active layer, with depth exhibiting the strongest correlations. Conversely, community composition and stochasticity ratio both displayed significant correlations with Eh, and had the strongest linkages with Tp in the permafrost layer (Figure 3b and Table S4). Additionally, bacterial richness was most closely and negatively related to depth, and the relative abundance of core taxa significantly influenced the stochasticity ratio in both the active and permafrost layers (Figure 3b). We also explored the environmental drivers of specific species with significant variations. Our results indicated that species exhibiting a significant decline in abundance along vertical profiles displayed strong sensitivity to variations in Tp, pH, POM, and TN, whereas those with increasing abundance showed weak environmental associations (Figure S4a). In the active layer, species (e.g., *Geobacter*) that decreased with degradation were significantly and positively correlated with PWC, while species insensitive to moisture (e.g., *Rubrobacter*) increased, suggesting that degradation might induce a taxonomic shift from anaerobic to drought‐tolerant taxa (Figure S4b). In contrast, both the species that proliferated or declined with degradation within the permafrost layer demonstrated weak responses to environmental changes (Figure S4c).

### 2.3 Co‐Occurrence Networks and Stability of Bacterial Communities

**FIGURE 4:**
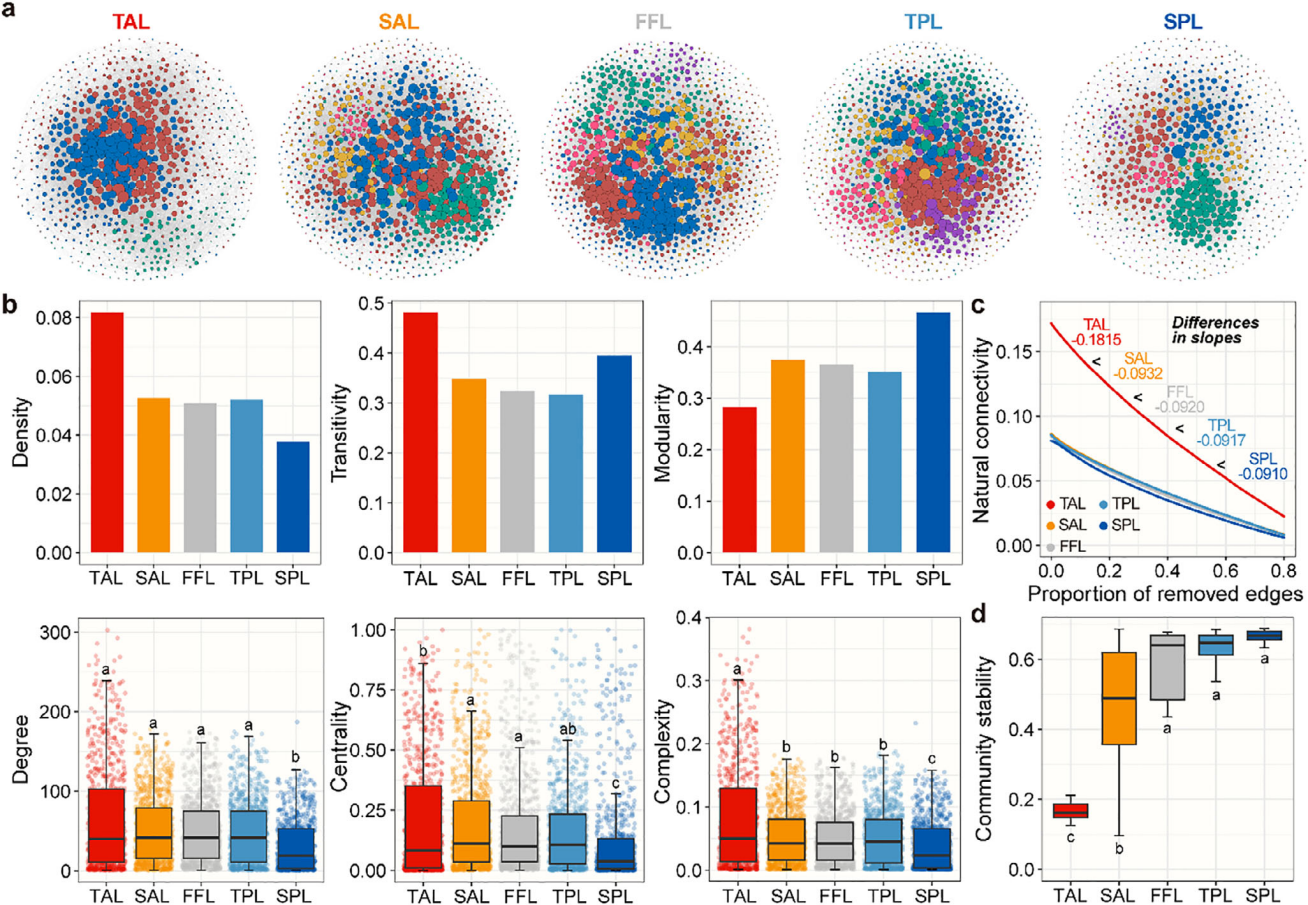
Co‐occurrence networks and stability of bacterial communities across the sub‐layers. (a) Nodes represent individual bacterial taxa whose size is positively correlated with the node degree; multiple node colors represent different modules. (b) Variations in network topological properties, including density, transitivity, modularity, degree, centrality (eigenvector), and complexity (linkage density; degree/node) across sub‐layers.(c) Robustness analysis exhibits the relationships between natural connectivity and the proportion of removed edges; larger changes in natural connectivity upon the same proportion of removed edges indicate less network robustness. (d) Variations in community stability calculated by the average variation degree method across sub‐layers. TAL, top‐active layer; SAL, sub‐active layer; FFL, frozen fringe layer; TPL, top‐permafrost layer; SPL, sub‐permafrost layer. Sample sizes of sub‐layers are *n* = 15, 31, 10, 49, and 20 for the top‐active layer (TAL), sub‐active layer (SAL), frozen fringe layer (FFL), top‐permafrost layer (TPL), and sub‐permafrost layer (SPL), respectively. Boxplots show median and interquartile range. Data are presented as mean ± s.e.m. Statistical significance is based on Kruskal–Wallis tests. Boxplots show median and interquartile range.

Bacterial co‐occurrence networks were constructed based on the SparCC algorithm to evaluate changes in possible ecological interactions among bacterial members and community stability across vertical profiles (Figure 4a and Figure S9a). We found notable decreases in the number of edges, density, transitivity, degree, centrality (eigenvector), and complexity (linkage density), alongside increases in modularity and proportion of positive edges across the main‐ and sub‐layers (Figure 4b and Figure S9b and Table S5), suggesting a shift toward more stable network structure [9]. This was further corroborated by the strengthened network robustness, as evidenced by the lesser degrees of declines in natural connectivity upon the removals of the same proportion of edges (Figure 4c and Figure S9c), also reflecting that bacterial communities became more stable [9, 45]. Consistent results were obtained using the average variation degree method [7], demonstrating significant increases in bacterial community stability (1 ‐ standardized average variation degree) through the main‐ and sub‐layers (Figure 4d and Figure S9d). Similarly, bacterial community stability significantly increased within the active layer (Figure S10a–d and Table S5), while not within the permafrost layer along the degradation gradient (Figure S11a–d and Table S5).

**FIGURE 5:**
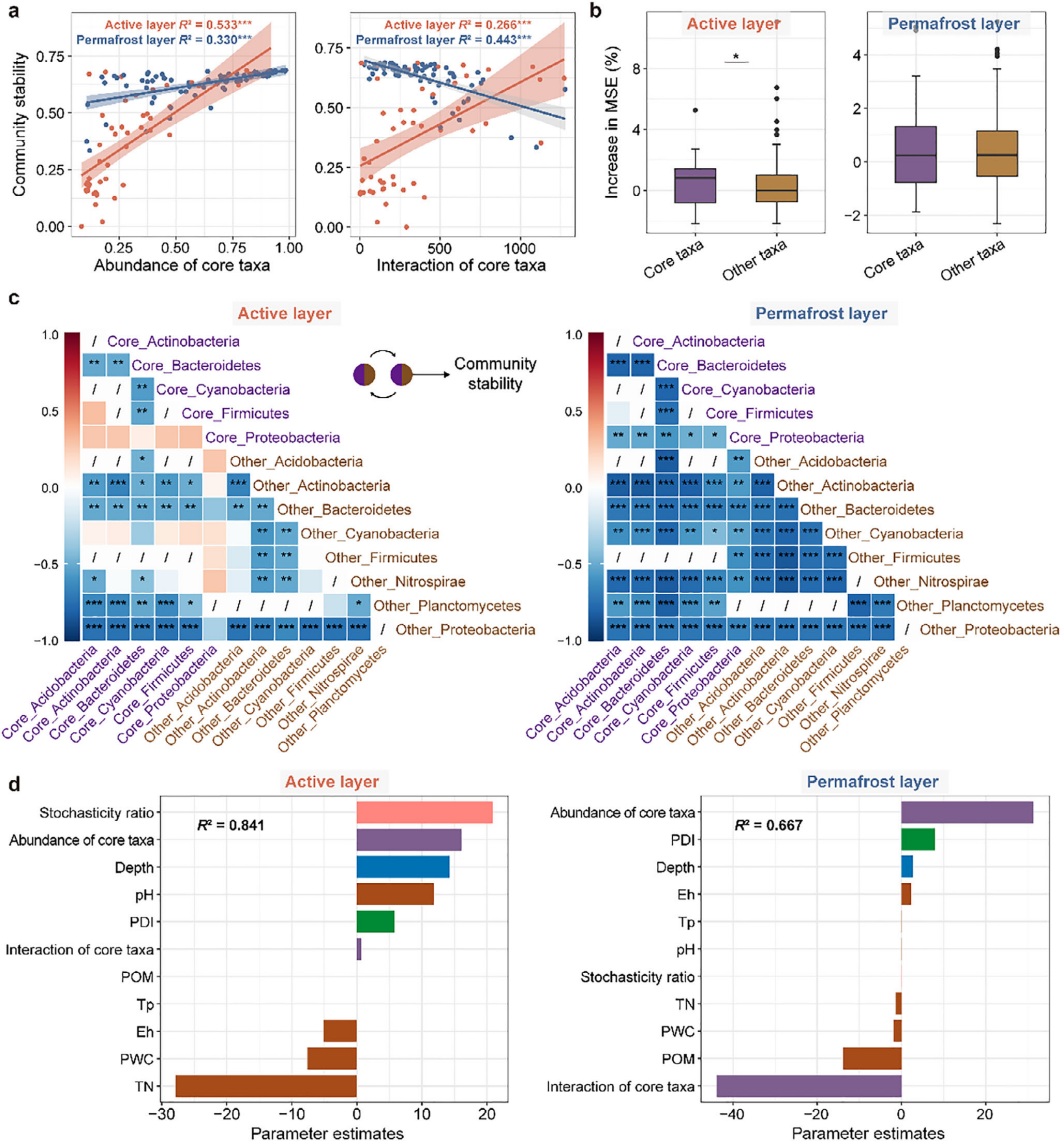
The roles of core taxa in affecting bacterial community stability. (a) Relationships of community stability with the relative abundance and interaction of core taxa in the active and permafrost layers. The interaction of core taxa is represented by the number of edges in the Single SparCC networks of core taxa. Sample sizes are *n* = 111 and 1639 in the active layer, and *n* = 111 and 569 in the permafrost layer for core and other taxa, respectively. (b) Importance of relative abundance of core and other taxa on predicting community stability using random forest model in the active and permafrost layers. (c) Correlations of the interactions among main core and other taxa with community stability in the active and permafrost layers. The interactions here are extracted from the SparCC networks constructed before. The color gradient indicates Spearman's rank correlation coefficients, with more positive values (dark red) indicating stronger positive correlations and more negative values (dark blue) indicating stronger negative correlations. For comparative purposes, only interactions between the phylum that both present in the active and the permafrost layers were selected for correlation analysis with community stability, so the slashes indicate that there are no interactions between the two phyla. Core_Phylum and Other_Phylum represent the core and other taxa in the corresponding phylum, respectively. (d) Relative importance of depth, PDI, permafrost properties (Tp, PWC, pH, Eh, POM, and TN), and core taxa (relative abundance and interaction of core taxa) on community stability in the active and permafrost layers as estimated by generalized linear models. The different colored columns represent different types of variables. The estimated relative importance corresponds to the standardized regression coefficients derived from generalized linear models. The magnitude of the value indicates the strength of the variable's influence, while the sign indicates the direction of the effect: positive values represent positive correlations, and negative values represent negative correlations. PDI, permafrost degradation index; Tp, permafrost temperature; PWC, permafrost water content; Eh, redox potential; POM, permafrost organic matter; TN, total nitrogen. Boxplots show median and interquartile range. Data are presented as mean ± s.e.m. Statistical significance is based on Kruskal–Wallis tests. Asterisks indicate statistical significance (***p < 0.001, **p < 0.01, and *p < 0.05).

We further explored the variables affecting bacterial community stability. We found that bacterial richness exhibited strong negative correlations with community stability in both the active and permafrost layers. However, the stochasticity ratio showed a positive association with community stability solely in the active layer (Figure S12). In addition, the relative abundance of core taxa was significantly and positively correlated with community stability in both the active and permafrost layers (Figure 5a). Networks of core taxa were constructed using Single SparCC network analysis, and the count of edges in each network served as a proxy for the interaction of core taxa. Despite the absence of remarkable variations in the interaction of core taxa across vertical profiles, this interaction significantly increased within the active layer as permafrost degraded (Figure S6d). Interestingly, the interaction of core taxa was strongly correlated positively with community stability in the active layer, but negatively in the permafrost layer (Figure 5a). Utilizing random forest models to compare the collective contributions of core taxa versus other taxa to community stability, we found that core taxa were the primary contributors in the active layer (Figure 5b). Subsequently, we quantified the interactions among core and other taxa within the major phyla. The strengths of these interactions were found to correlate more significantly and strongly with community stability in the permafrost layer than in the active layer (Figure 5c). In particular, the correlations of community stability with the interactions between core taxa in Proteobacteria and other core taxa shifted from non‐significant positive in the active layer to significant negative in the permafrost layer (Figure 5c), suggesting the essential roles of these interactions in affecting community stability in the permafrost layer. In addition, generalized linear models revealed that TN, stochasticity ratio, and relative abundance of core taxa were the important drivers of community stability in the active layer. Whereas in the permafrost layer, the interaction of core taxa emerged as a key determinant (Figure 5d), which could be attributed to the shorter path length and stronger betweenness of edges among core taxa (Figure S13). This is because, based on theoretical expectations, these variations could make the communities collapse more rapidly and extensively when networks face disturbances (see details in *Experimental Section*) [46, 47, 48].

### 2.4 Links Among Environmental Variables, Bacterial Community Attributes, and Permafrost C Storage

**FIGURE 6:**
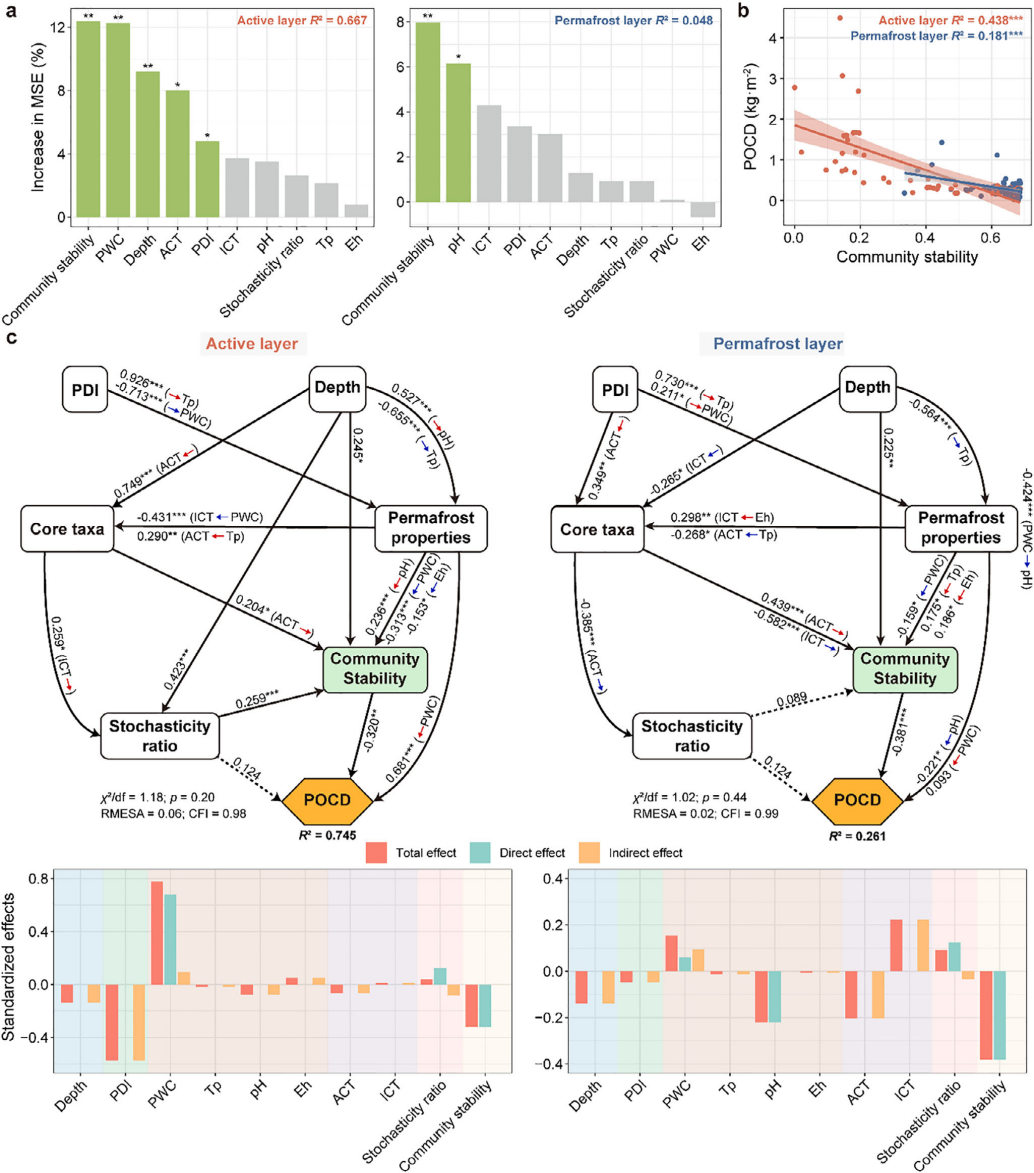
Effects of environmental variables and bacterial community attributes on permafrost organic C storage (POCD). (a) Importance of depth, PDI, permafrost properties (Tp, PWC, pH, Eh), stochasticity ratio, core taxa (ACT and ICT), and community stability on predicting POCD using random forest model in the active and permafrost layers. (b) Relationships of community stability with POCD in the active and permafrost layers. (c) Structural equation models showing the effects of depth, PDI, permafrost properties, stochasticity ratio, core taxa, and community stability on POCD in the active and permafrost layers. We grouped the different categories of predictors (permafrost properties and core taxa) into the same boxes in the model for graphical simplicity. However, these boxes do not represent latent variables. Among boxes, the black solid lines represent significant paths, and the black dashed lines represent non‐significant paths. Among variables, red arrows represent positive paths and blue arrows represent negative paths. Standardized effects are categorized as follows: direct effects represent the direct influence of one variable on another; indirect effects represent the influence mediated through intermediate variables; and total effects represent the sum of direct and indirect effects. *R* ^2^ represents the proportion of variance explained for POCD. A large *p*‐value indicates that the predicted model and observed data are equal, that is, good model fitting. Below is the standardized total, direct, and indirect effects on POCD derived from models. Sample sizes are *n* = 51 and 74 for the active and permafrost layers, respectively. POCD, permafrost organic carbon density; PDI, permafrost degradation index; Tp, permafrost temperature; PWC, permafrost water content; Eh, redox potential; POM, permafrost organic matter; TN, total nitrogen; ACT, relative abundance of core taxa; ICT, interaction of core taxa. Asterisks indicate statistical significance (***p < 0.001, **p < 0.01, and *p < 0.05).

Permafrost C storage, represented by permafrost organic C density (POCD) [9], was significantly higher in the active layer than in the permafrost layer, with a considerable reduction from the top‐active layer to the sub‐active layer, but no marked variations from the sub‐active layer to the sub‐permafrost layer. Along the degradation, permafrost C storage showed a noticeable decline within the active layer, with a greater loss than the permafrost layer, which could reach up to 70.2% (Figure S14). Notably, bacterial community stability played the most important role in affecting permafrost C storage in both the active and permafrost layers (Figure 6a). Bacterial community stability was significantly and negatively correlated with POCD across vertical profiles, with the highest effect in the active layer (Figure 6b and Figure S15). As permafrost degraded, this negative correlation became more significant and intense (Figure S15). We further examined the profiles of carbohydrate‐active enZymes (CAZymes) and found that the abundances of genes encoding enzymes for C degradation were strongly negatively correlated with community stability in both the active and permafrost layers, as well as across vertical profiles (Figure S16). This functional loss involved enzymes targeting both labile (e.g., starch) and recalcitrant (e.g., lignin and chitin) C, suggesting that the highly stable communities induced by permafrost degradation might lose the enzymatic capacity to transform organic matter into soil C. Structural equation modeling (SEM) was further utilized to investigate the effects of environmental variables and community attributes on permafrost C storage (Figure 6c). We found that community stability was negatively affected by PWC, which decreased due to PDI in the active layer but increased in the permafrost layer. Eh showed a negative impact on community stability, but the opposite in the permafrost layer. Moreover, community stability was negatively linked to POCD in both the active and permafrost layers, even when considering other variables simultaneously. In the active layer, 74.5% of the variance in POCD was explained, with PWC exerting the most substantial positive effect. Differently, 26.1% of the variance in POCD in the permafrost layer was explained, with community stability playing a predominant role in governing POCD. PDI displayed a strong and negative effect on POCD in the active layer, and a weak but still negative impact on POCD in the permafrost layer (Figure 6c), showing that permafrost degradation undoubtedly caused C loss.

To validate the importance of bacterial communities in permafrost C storage, we performed machine learning‐based methods, including bagged regression tree and random forest models. Both models explained the high variances in permafrost C storage and showed the low mean squared error (MSE) and root mean squared error (RMSE) (Figure S17a). The models trained by datasets S1, S2, and S3 also captured effectively the changes in permafrost C storage in datasets S4 and S5 (Figure S17b), confirming the predictive power of bacterial communities for permafrost C storage. Among the bacterial predictors, the 20 most important taxa for POCD included methane‐cycling taxa (e.g., *Methylomonas methanica*) and nitrogen (N)‐fixing bacteria (e.g., *Mesorhizobium ciceri*), suggesting that C dynamics in permafrost are tightly linked to C–N coupling mechanisms (Figure S18).

## 3 Discussion

Consistent with our hypothesis, we found that bacterial community stability increased from the active layer to the permafrost layer, as evidenced by the weakened environmental sensitivity, promoted network stabilizing properties, strengthened robustness, and reduced average variation degree, which align with previous studies [7, 9, 10]. These findings can be explained by the observations that frequent freeze–thaw processes in the active layer introduce environmental fluctuations (e.g., temperature, water, and salinity) [49], which attenuate dispersal limitation and act as filtering stress to stimulate species turnover in microbial communities, thus leading to relatively high deterministic processes [28, 29]. This deterministic assembly was substantiated by the strong coupling between specific species and environmental variables found in our study. For instance, anaerobic genera, such as *Geobacter* and *Terriglobus*, exhibited strong positive correlations with PWC and sharply declined with degradation‐induced aridification, confirming that environmental filtering exerts a stringent selection pressure. Although the weakening of the deterministic processes with depth was consistent with observations in Alaskan soils, a key distinction was that environmental change was the main driving factor there [50, 51], whereas depth was the dominant driver in our study. This discrepancy likely stems from the intense cryoturbation in Arctic tundra, which disturbs vertical layers and decouples depth from environmental gradients [52]. Conversely, the strict vertical stratification in alpine permafrost allows depth to act as a composite proxy for the linear intensification of environmental constraints. Moreover, the release of nutrients facilitates the creation of more ecological niches and amplifies the interactions within communities, which propel microbial diversification but reduce community stability [8, 28].

In contrast, the permafrost layer largely acts as long‐term cold storage, and microbial communities here display adaptive strategies to sub‐zero, oligotrophic, and high‐salinity conditions (e.g., via increased membrane fluidity and the production of cold and osmotic stress proteins) to maintain high stability [23]. The low metabolic activity and high dormancy of species, combined with the precluded water movement in the frozen state, can exacerbate dispersal limitations [28, 53], which could also explain the higher spatial heterogeneity observed in the permafrost layer [30]. Although stress‐tolerant species like *Acinetobacter junii* accumulated in the permafrost layer, they showed weak associations with nutrients and permafrost properties. This decoupling suggests that community composition is not driven by recent niche selection, but rather reflects a legacy of geologic‐scale environmental filtering that has shaped the reduced communities observed today [54, 55, 56]. Consequently, the persistence of these ancient and dormant lineages leads to assembly patterns driven more by stochasticity, and the primary mechanism for niche development and diversity increase in the permafrost layer is likely through drift [28, 57]. In this context, the taxonomic composition exhibits notable regional distinctness. While the permafrost layer in older Arctic permafrost was typically characterized by an enrichment of spore‐forming Firmicutes [58, 59], the permafrost layer in our study was consistently dominated by Proteobacteria. This divergence implies that regional geological legacies shape distinct microbial seed banks, rather than a uniform ancient community [59]. Due to the lower and more homogeneous availability of substrates [37], and the relatively unaltered environmental conditions at greater depths, microorganisms do not change much from the frozen fringe layer to the sub‐permafrost layer. This pattern also mirrors observations in the Arctic tundra, where the microbiome composition within the frozen fringe layer remains statistically similar to that of the permafrost layer, indicating that the intermittent thaw has not yet induced a shift toward the active layer microbes [60]. Furthermore, core taxa have been widely proven to play an essential role in maintaining community stability [38, 61, 62]. Interestingly, the interaction of core taxa emerges as the most pivotal predictor for community stability in the permafrost layer, which could be attributed to the fact that responses of core taxa to environmental disturbances are propagated to other taxa via ecological interactions [63]. As demonstrated in this study, since these responses are propagated more rapidly among core taxa, whose interactions mediate the most transmission pathways, an increase in the interaction of core taxa may expose more taxa to the ripple effects of disturbances, thereby accelerating the collapse of communities [46, 47, 48].

Our previous work showed that permafrost degradation reduced bacterial community stability in the active layer [9]. However, we found that bacterial community stability was enhanced in the active layer as degradation increased. In fact, the five sampling sites could be categorized as transitional permafrost (S1) and severely‐degraded permafrost (from S2 to S5) in line with the permafrost degradation stages classified by our previous research [9]. Expanding on the earlier discoveries, we hypothesized that community stability would substantially decrease during the lightly‐degraded stage, reach a turning point during the transitional stage, and slightly increase during the severely‐degraded stage, with an overall higher level in the lightly‐degraded stage than in the severely‐degraded stage (Figure S19). This hypothesis was supported by our finding that community stability was not higher at S2 than S1 within the active layer. It implies that bacterial communities can develop adaptations and resilience in response to constant environmental stress [64, 65], and cause a rebound in community stability [64]. Additionally, the discrepancy with previous findings could also be attributed to seasonal dynamics. The previous study primarily focused on summer samples when microbial metabolic activity and competition were high, making community networks more susceptible to destabilization by degradation disturbance. In contrast, our sampling in the fall captured communities transitioning toward dormancy and cold adaptation. This seasonal shift might promote the formation of stress‐tolerant co‐occurrence patterns, thereby exhibiting higher stability compared to the summer communities [66]. Consequently, this strong seasonal dependence underscores the critical need for future longitudinal monitoring to fully disentangle transient seasonal fluctuations from persistent degradation‐driven shifts in community stability. Previous studies have indicated that the enhancement of stability is linked to increases in the population of abundant species (e.g., core taxa) [19] and stochastic processes [67], as well as reductions in community diversity [19] and interactions [68]. The same phenomena were also observed in our study, which comprehensively reveals the responses of microbial communities to permafrost degradation in the active layer. However, these microbial variations did not occur in the permafrost layer, suggesting that microbial communities held lower sensitivity to degradation compared to those in the active layer in alpine permafrost during fall.

Permafrost degradation, whether in the active or permafrost layers, could exacerbate C loss. Interestingly, bacterial community stability showed a negative association with permafrost C storage. While stable communities are often beneficial to C sequestration, our finding aligns with a previous study indicating that stable community structure could aid in C decomposition [69], owing to the preservation of the original “functionally optimal” community structure maximized by stable communities, which can fully exploit the available resources [69, 70]. Furthermore, a few studies have shown that the increased adaptation (i.e., enhanced stability) of microbial communities to warming can exacerbate C loss [69, 71, 72]. Likewise, we demonstrated that the same would happen if communities adapted to permafrost degradation. However, some studies have found that microbes could reduce their physiological activity to increase acclimatization, thereby reducing their respiration and C loss [73, 74, 75]. Previous research has reported that permafrost degradation can alter the water and redox states, while the stimulated microbial activity may increase the abundance of methanogenic and C decomposition genes to accelerate C emissions [76, 77]. In our study, we identified a loss of metabolic potential of CAZymes that was associated with high community stability, implying that the C loss is linked to an impaired microbial capacity to assimilate and transform organic matter, which limits the replenishment of the soil C pool. Meanwhile, the relationship between community stability and C storage was stronger in the active layer. Consequently, the ecological consequences of degradation‐induced C loss, such as the destabilization of the soil C pool, disruption of ecosystem elemental cycling, and positive feedback to climate warming, are likely far more severe in the active layer than the permafrost layer.

Taken together, this study provides a valuable snapshot for understanding bacterial community stability and its link to carbon storage across vertical profiles and degradation gradient in alpine permafrost, offering a pivotal baseline for future time‐series studies. However, several limitations should be acknowledged to frame future research directions. First, our study used a methodology of space‐for‐time analysis, relying on single time‐point sampling from the QTP during the fall. Given that key environmental variables of permafrost (e.g., thaw duration, temperature, and moisture) are highly spatially heterogeneous and temporally dynamic, it is necessary to conduct longitudinal and cross‐seasonal monitoring to directly track the variations in microbial communities under permafrost changes, alongside large‐scale validation to generalize these patterns to high‐latitude Arctic regions. Supplementing non‐ or lightly‐degraded permafrost samples would also help deepen our understanding of permafrost microbial dynamics. Second, since bacteria are the dominant decomposers in many soil systems, our current analysis focused exclusively on the bacterial kingdom. Other microbial taxa, including archaea, fungi, and viruses, also play critical roles in permafrost biogeochemical cycling and C turnover. Their responses to degradation and interactions with bacteria remain an important knowledge gap that warrants comprehensive investigation in future multi‐kingdom studies. Finally, from a methodological perspective, we utilized read‐based metagenomic profiling (Kraken2) to maximize the data utilization rate for diversity analysis and network constructions. While this approach provides a robust overview of the total community structure, it lacks the fine‐scale resolution of genome‐resolved metagenomics (i.e., Metagenome‐assembled genomes, MAGs). We acknowledge that assembling MAGs would offer deeper insights into specific metabolic pathways and the functional potential of uncultured lineages. Therefore, future work should prioritize genome‐centric analyses to elucidate the link between specific microbial responders and the functional genes driving permafrost C loss. Nevertheless, given the important value of bacterial communities in effectively predicting permafrost C storage, we can translate this knowledge into viable strategies (e.g., microbial inoculation and compound addition [78, 79] for managing microbial community systems for minimizing C loss in alpine permafrost.

## 4 Conclusion

Understanding how microbial communities and their associations with C storage across vertical profiles respond to permafrost degradation is a critical topic in permafrost microbial ecology. By examining variations of bacterial community attributes in the five 15 m‐depth permafrost cores, this study provided clear evidence that the active layer hosted the higher bacterial *α*‐diversity, while the communities in the permafrost layer exhibited higher stochastic processes and stability. Moreover, these community attributes varied significantly from the top‐active layer to the sub‐active layer and then exhibited non‐significant changes from the frozen fringe layer to the sub‐permafrost layer. As permafrost degraded, bacterial *α*‐diversity decreased, but the stochastic processes and community stability increased within the active layer. Taxonomically, this degradation‐induced response was marked by a distinct succession from anaerobic taxa (e.g., *Geobacter*) to drought‐tolerant taxa (e.g., *Rubrobacter*). However, these attributes within the permafrost layer were not affected by degradation. We also found that core taxa played an important role in maintaining community stability, with their relative abundance showing large contributions in the active layer, while their interaction predominantly drove community stability in the permafrost layer. Importantly, in the context of permafrost degradation, we observed a negative association of bacterial community stability with C storage, while this relationship was stronger in the active layer than in the permafrost layer. Coupled with the observed functional depletion of C‐degrading genes, these results reveal that permafrost degradation may lead to an increase in microbial‐mediated C loss, potentially causing positive climate feedback under future warming.

## 5 Experimental Section

### 5.1 Site Description

The sampling sites are located in the Shule River headwaters (38.0 to 39.0°N, 97.3 to 99.0°E, and 3402 to 5814 m above sea level) on the western part of Qilian Mountains, the northeast margin of the QTP, China. According to the meteorological data of the past 10 years (unpublished data), the annual mean air temperature and precipitation range from −2.9°C to −4.2°C and 331 to 561 mm, respectively. Approximately 68.40% of the Shule River headwaters is covered by alpine grassland, whose main vegetation types include alpine swamp meadow, alpine meadow, and alpine steppe [80]. The dominant plant species include *Kobresia tibetica*, *Stipa purpurea*, *Kobresia pygmaea*, and *Poa pratensis* [81]. According to the World Reference Base for Soil Resources, the soil types include Calcisols, Umbrisols, and Gleysols [82]. Permafrost is extensively developed and occupies about 97.99% of this region [83].

### 5.2 Sample Collection and Analysis

In early October 2020, we collected samples from 15 m‐depth permafrost cores by drilling at five distinct sites (Figure 1a and Table S1). Specifically, we used a drilling rig and drove stainless steel tubes into the permafrost without using drilling fluids to retrieve the intact permafrost cores. We used sterilized scalpels to remove the outer 1 cm of each core surface to eliminate potential contamination. First, we collected the samples for metagenomic sequencing into the cryotubes and placed them in a liquid nitrogen tank for transfer to the laboratory for frozen storage at −80°C. Second, we used a cutting ring (volume of 100 cm^3^) to collect the samples for measuring bulk density and PWC. Finally, the samples for the determination of pH and Eh were collected and stored at 4°C, while the samples for the measurement of POC and TN were collected and air‐dried. Notably, all parts of the samples were collected at 10 cm intervals, for a total of 150 samples of each type per core (Figure 1b).

Tp from 0.1 to 15 m in the five boreholes were automatically monitored using Hydra Probe II soil sensors (Stevens) and the temperature chains equipped with thermistors developed by the State Key Laboratory of Frozen Soil Engineering (SKLFSE, China), connected to the CR1000X and CR6 datalogger (Campbell Scientific), respectively. Based on the active layer thickness calculated by the monitoring data of Tp [84], the permafrost vertical profiles at the five sampling sites were consistently divided into two main‐layers including the active and permafrost layers, and five sub‐layers containing top‐active layer, sub‐active layer, frozen fringe layer, top‐permafrost layer, and sub‐permafrost layer (Figure 1c). Specifically, the top‐active layer is defined as the layer from 0 to 0.5 m depth; the sub‐active layer ranges from 0.5 m depth to the bottom‐active layer; the frozen fringe layer extends from the bottom‐active layer to the table‐permafrost layer (the zone 20 cm above and below the junction of the active and permafrost layers); the top‐permafrost layer refers to the layer from the table‐permafrost layer to 12 m depth, where 12 m is the approximate average depth of zero annual amplitude (the depth at which the annual range of Tp is equal to 0.1°C or the adjacent depth) [85] in our sampling region (unpublished data); and the sub‐permafrost layer spans from 12 to 15 m depths. Finally, 25 samples from each intact permafrost core at specific depths were selected for subsequent metagenomic sequencing and physicochemical properties analysis (Table S6). POC and TN were measured through the Walkley–Black dichromate oxidation method and micro‐Kjeldahl procedures, respectively. POM and POCD were calculated as previously described [9]. To determine pH and Eh, composite electrodes were connected to a PHBJ‐260 pH meter (INESA) with water: soil ratio of 5: 1. PWC was determined based on the mass differences of fresh samples after drying at 105°C for 24 h.

### 5.3 Sequencing and Bioinformatics

DNA extraction was performed on the thawed samples using a modified cetyl trimethyl ammonium bromide (CTAB) method. After rinsing with phosphate‐buffered saline (PBS) buffer and removing gravel, 3 g of the processed soil per sample was weighed for extraction. The sample suspension was homogenized using a tissue homogenizer, followed by centrifugation at 12 000 rpm for 5 min. The resulting supernatant was discarded, and the remaining material was resuspended in the Tris‐EDTA (TE) buffer. The lysis process was achieved using a preheated CTAB/NaCl solution at 65°C. DNA extraction was conducted using a phenol: chloroform: isopentanol (25: 24: 1) mixture, followed by precipitation with isopropanol. Purification of the DNA was carried out using 1X AMPure XP beads (VAZYME Biotech, China). Subsequently, the DNA underwent whole genome amplification (WGA) using random hexamer (N6) primers (VAZYME Biotech, China) and Phi29 DNA polymerase (VAZYME Biotech, China). Finally, shotgun metagenomic sequencing libraries were constructed using a previously established method [86], which is a high‐throughput library preparation technique ensuring low bias. The paired‐end metagenomic sequencing was conducted on the DNBSEQ‐T7 platform (MGI, China) with 100 bp read length. Quality control of raw reads was performed using fastp (v0.23.2) to filter low‐quality sequences and adapters [87]. The sequencing data exhibited high quality and throughput across all 125 samples. On average, we obtained 41.1 gigabases (Gb) clean reads per sample, with an average Q30 value of 91.1% (Table S7). To obtain a comprehensive profile of the microbial community, we employed a read‐based classification approach rather than assembly‐based methods. While MAGs can provide valuable metabolic context, they often exclude a significant portion of reads, particularly from low‐abundance populations [88]. Given our study focused on community‐wide attributes (e.g., diversity, stochasticity, and stability) and co‐occurrence networks, the read‐based approach ensures a more holistic representation of the community structure. Therefore, taxonomic classification was performed using Kraken2 with PlusPFP (date 2021/01/27) using default parameters [89]. Kraken2 utilizes exact k‐mer matches to assign taxonomy to individual reads, maximizing data utilization. The relative abundance of taxa was further re‐estimated using Bracken to correct for genome length and read distribution [90]. Notably, since the Kraken‐based method only annotated a small number of archaea, we focused our analysis on bacteria. To correct for uneven sequencing depth, our dataset was rarefied to the minimum number of reads per sample using the “*rarefy_even_depth*” function in the “*phyloseq*” R package before calculating *α*‐ and *β*‐diversity and subsequent analysis [91]. Based on the obtained species abundance and annotation tables, the 10 bacterial phyla with the highest relative abundances were defined as the major phyla, and the remaining phyla were combined as “Others”. Furthermore, the high‐quality reads were assembled using MEGAHIT (v1.2.9) with default parameters, and contigs exceeding 500 bp were retained [92]. These assembled contigs were utilized as input for gene prediction using Prodigal (v2.6.3) [93]. The predicted genes were clustered into a non‐redundant gene catalog using the mmseqs2 (v14.7e284) easy‐cluster pipeline, employing thresholds of 80% coverage and 90% identity [94, 95]. The non‐redundant genes were functionally annotated against the CAZyme database using dbCAN3 with default parameters [96].

### 5.4 Statistical Analysis

Bacterial richness and Shannon index were measured using the “*vegan*” R package to quantify *α*‐diversity [97]. *β*‐diversity based on Bray‐Curtis distance coupled with PCoA was calculated to indicate bacterial community dissimilarity, differences among layers and sites were tested by PERMANOVA with the “*vegan*” R package [97]. We also calculated the distances of bacterial communities to their group centroids, and their differences in values indicate community dispersion or heterogeneity among groups. Since bacterial richness was highly correlated with the Shannon index in both the active layer and permafrost layers, it was used to represent bacterial *α*‐diversity in subsequent analyses (Figure S20). Pearson correlations among environmental variables (depth, PDI, and permafrost properties) and their associations with *α*‐diversity were calculated and visualized using the “*Hmisc*” and “*corrplot*” R packages [98, 99], respectively. Before the analysis, we performed a transformation on these variables to ensure they met the statistical assumptions. Euclidean distances based on the matrix of measured environmental variables were calculated to fit with community similarity. Specifically, PDI refers to the first component of principal component analysis for active layer thicknesses and MAGTs using the “*stats*” R package, which was used to rank the degraded degree of the five permafrost sampling sites, in ascending order from S1 to S5 (Figure 1c). Importantly, we adopt a methodology of space‐for‐time analysis, considering spatial patterns of different degraded permafrost sampling sites as a temporal series of a gradient of permafrost degradation [9].

Null model analysis was carried out using the “*NST*” R package to evaluate the importance of stochastic processes in bacterial community assembly [100], which was quantified by dividing the mean expected similarity in the null model by the observed similarity [101]. The boundary of the stochasticity ratio is 0.5 to distinguish between assemblies that are more deterministic (< 0.5) and those that are more stochastic (> 0.5). The neutral community model was parameterized to estimate the migration rate by using the “*minpack.lm*” R package [102]. Niche overlap was determined according to Levin's niche overlap index with the “*spaa*” R package [103]. To evaluate the affecting variables of bacterial composition and stochasticity ratio, a modified Mantel test with linear models was performed [104]. Specifically, we performed logarithmic transformation or not on community composition, stochasticity ratio, and each environmental variable, and then fitted four models for each variable pair, including Y∼X, Y∼ln(X), ln(Y)∼X, and ln(Y)∼ln(X). The best models were chosen for the results of Mantel test. When performing a logarithmic transformation on a factor with zero or negative values, all values were subtracted by the minimum value before applying the transformation. For zero values obtained after the subtraction, they were substituted with the 0.05 of minimum positive value (i.e., ‐3.00 in the natural log) prior to the transformation [104].

The SparCC algorithm was employed to construct bacterial co‐occurrence networks by the “*SpiecEasi*” R package [105]. Given the variability in sample numbers across different layers or sites, the construction of networks involved aligning the number of samples in each layer or site with the minimum sample count. This alignment was achieved by averaging the bacterial abundance data of samples with adjacent depths based on the depth information of each sample. This adjustment ensured that different layers or sites had the same number of samples to ensure networks were comparable. The SparCC results were filtered by the thresholds *r* > 0.35 and *p* < 0.05 for different layers and by the thresholds *r* > 0.65 and *p* < 0.05 for different sites. The networks were visualized using Gephi platform. Network properties including density, transitivity, modularity, degree, centrality (eigenvector), and complexity (linkage density; degree/node) were calculated by the “*igraph*” R package [106]. Previous studies have concluded that lower density, transitivity, degree, centrality, and complexity, but higher modularity, represent higher network stability [9]. Network robustness was tested by attacking edges on natural connectivity [9], and is considered as an indicator of microbial community stability [45]. The average variation degree method was used to directly assess bacterial community stability (1 ‐ standardized average variation degree), and this method is not limited by sample size and has advantages in assessing community stability over other methods [7]. Combining network and average variation degree methods to assess community stability has been widely used [107].

Previous studies used different criteria to identify core taxa [108], so in this study we referred to other studies and selected the top 5% bacterial species‐level taxa with the highest mean relative abundance and the frequency of occurrence in more than 70% of all samples, resulting in a set of 111 taxa that were defined as core taxa [108, 109]. Bacterial taxa excluding the core taxa represented other taxa. Bacterial interaction was represented by the count of edges among taxa in networks. Additionally, we extracted the subnetworks of the individual samples from the SparCC networks to count the numbers of edges among major phyla of core and other taxa (Table S8), and their Spearman correlations with community stability were calculated and visualized using the “*Hmisc*” and “*corrplot*” R packages [98, 99], respectively. The path length and edge betweenness among nodes of core taxa were also extracted using the “*igraph*” R package [106]. A shorter average path length can lead to faster information dissemination and higher efficiency within the network, while edge betweenness represents the frequency with which an edge is used as the shortest path in a network. Based on theoretical expectations, when the network faces disturbances, these properties cause the disturbance signals to propagate faster and more widely to the nodes, and the responses generated by these nodes can cause the network to collapse rapidly and more extensively, ultimately resulting in a loss of stability [46, 47, 48]. These two properties can help us estimate the role of the interaction of core taxa in information dissemination and community stability. Since the extracted results comprised 50 samples, which did not encompass the entirety of individual samples (*n* = 125 samples), we further constructed the Single SparCC networks specifically for core taxa to obtain the edge information of all samples (Table S9), and the detailed methodology can be found in a prior study [110]. This approach ensured that our subsequent analyses (e.g., SEM) continued to use the original data rather than the averaged data, enhancing the credibility of our findings. By extracting the interaction of core taxa from SparCC networks and fitting them to the averaged data of community stability in the active and permafrost layers, we obtained results consistent with those derived from the Single SparCC network analysis (Figure S21). This demonstrated the feasibility of the Single SparCC network analysis and reinforced the accuracy of our results.

We employed the generalized linear models to assess the relative influence of environmental variables, core taxa (relative abundance and interaction of core taxa), and stochasticity ratio on community stability. As bacterial richness was highly correlated with community stability in both the active layer and permafrost layers (Figure S18), it was excluded from the models, but this did not mean that we neglected the impact of richness on community stability. The quantitative comparisons of the relative influence of each variable were conducted through standardized model coefficients, and the total contribution of each model was expressed as the percentage of explained variance [111]. Generalized linear models were conducted using the “*h2o*” R package [112].

The main bacterial predictors of community stability were identified by random forest models using the “*randomForest*” R package [113], and species‐level taxa with the relative abundance greater than 0.01% among core and other taxa were used as predictors, respectively. The relative importance of environmental variables, core taxa, and stochasticity ratio on POCD were also calculated by random forest models. Consistent with the previous analysis, richness was excluded as a predictor variable due to its high correlation with community stability. Before performing the models, we also removed TN and POM from permafrost properties due to their strong collinearities with POCD (*R* ^2 ^> 0.5) (Figure S22). We utilized ntree = 1000 to ensure the convergence of importance estimates and the default mtry parameter. The relative importance of predictors was evaluated based on the increase in mean squared error (MSE). Bagged regression tree and random forest models were performed to predict POCD using the “*gbm*” and the “*randomForest*” R packages [113, 114], respectively. We used bacterial communities (species‐level taxa abundance) as predictors and POCD as the response variable. 70% of the dataset was randomly separated into a training set, with the remaining 30% used as a testing set. To optimize model performance and avoid overfitting, hyperparameter tuning was conducted using 10‐fold cross‐validation. The optimal parameters (e.g., mtry for random forest model and interaction.depth for bagged regression tree) were selected using the “*caret*” R package [115]. To further validate the predictive ability of bacterial communities on POCD, we also selected the datasets of S1, S2, and S3 as a training set to test the datasets of S4 and S5. We used MSE and RMSE to evaluate the machine learning models obtained from the testing set, with their lower values indicating better prediction performance of models. Also, the increase in MSE from the random forest model was used to identify the top 20 species that were most important to POCD.

The impacts of bacterial community stability were assessed by linear mixed‐effects models using the “*lme4*” R package [116]. In the models, community stability was termed as a fixed effect. When we tested these impacts in the active or permafrost layers, the sampling site was termed as a random intercept effect. Similarly, when we tested these impacts across the five sites (along the gradient of permafrost degradation), the sub‐layer was termed as a random intercept effect. Effect sizes were represented by the regression coefficients in the models. Wald type II *χ* ^2^ tests were used to calculate the *p* values from the models using the “*car*” R package [117].

SEM was performed to determine the effects of the environmental variables, core taxa, stochasticity ratio, and community stability on POCD. As described previously, we removed TN and POM from permafrost properties due to their strong collinearities with POCD before performing SEM. We considered a comprehensive model encompassing all plausible pathways and then excluded non‐significant and biologically meaningless pathways in a sequential manner [118]. The procedure was iterated until the model achieved a satisfactory fitting, indicated by *p* values of *χ* ^2^ test > 0.05 and the root mean square error of approximation < 0.08. The corresponding analysis was conducted using the “*lavaan*” R package [119].

Regarding the other analysis, the first component of principal component analysis for active layer thicknesses and MAGTs was calculated to represent PDI using the “*stats*” R package. We used Kruskal–Wallis tests (with a Benjamini & Hochberg's correction) to compare permafrost properties, POCD, bacterial community attributes between two main‐layers, among five sub‐layers, and among five sites in the active and permafrost layers by the “*stats*” R package (p.adjust.method argument set to “BH”) [120]. In addition, the percentage change in permafrost C storage was obtained by subtracting the POCD of the previous group from that of the next group and dividing by that of the previous group. To identify specific taxonomic responders associated with vertical profiles and permafrost degradation, we performed the linear trend analysis on relative abundances at the species level. First, species with an average relative abundance below 0.01% were filtered out to reduce noise. We then used the “*Hmisc*” R package to calculate the Spearman's correlation coefficient (r) between the relative abundance of each species and the continuous gradients of vertical profiles (from top‐active layer to sub‐permafrost layer) or permafrost degradation (from S1 to S5) [98]. The top 20 species exhibiting the strongest linear responses (ranked by the absolute value of r) were selected as indicator species for each gradient. To explore the environmental drivers of these indicators, Spearman's correlations were calculated between their relative abundances and environmental variables using the “*Hmisc*” R package [98].

## Funding

This work was supported by the National Key R&D Program of China (Grant No. 2022YFF0801903), “Light of the West” Cross‐team Project of the Chinese Academy of Sciences (Grant No. xbzg‐zdsys‐202214), the National Natural Science Foundation of China (Grant Nos. U23A2062 and U24A20586), the Science and Technology Program of Gansu Province (Grant No. 23ZDFA017), and the Freedom Project of the State Key Laboratory of Cryospheric Science and Frozen Soil Engineering, Northwest Institute of Eco‐Environment and Resources, CAS (Grant No. CSFSE‐FX‐2505).

## Conflicts of Interest

The authors declare no conflicts of interest.

## Supporting information




**Supporting File 1**: advs74402‐sup‐0001‐SuppMat.docx.

**Supporting File 2**: advs74402‐sup‐0002‐SITables.xlsx.

## Data Availability

The data that support the findings of this study have been deposited into CNGB Sequence Archive (CNSA) of China National GeneBank DataBase (CNGBdb) with accession number CNP0004487 (https://db.cngb.org/search/project/CNP0004487/). The custom code and scripts used for statistical and microbiome analyses have been made publicly available at Zenodo (https://zenodo.org/records/18050666). The meteorological data are available from the corresponding author upon reasonable request.
