# Supplementary material for: Divergent Responses of Bacterial Communities to Permafrost Degradation and Their Associations With Carbon Across Vertical Profiles
Source: Adv Sci (Weinh). 2026 Feb 15;13(23):e10516. doi: 10.1002/advs.202510516 (PMC13104071; doi:10.1002/advs.202510516)
Supplement: Supplementary file 1 — Supporting File 1: advs74402‐sup‐0001‐SuppMat.docx. [file ADVS-13-e10516-s001.docx]

Supporting Information

**Divergent Responses of Bacterial Communities to Permafrost Degradation and Their Associations with Carbon Across Vertical Profiles**

Shengyun Chen*, Yuzheng Gu, Ali Bahadur, Enyan Liu, Tonghua Wu, Xiaofan Zhu, Yuanqiang Zou, Hewei Liang, Peijie Wei, Linwei Wu, Qingbai Wu, Peizhi Yang, Hongyan Yu, and Yunfeng Yang

*^*^Corresponding author*

*Corresponding author E-mail: sychen@lzb.ac.cn*


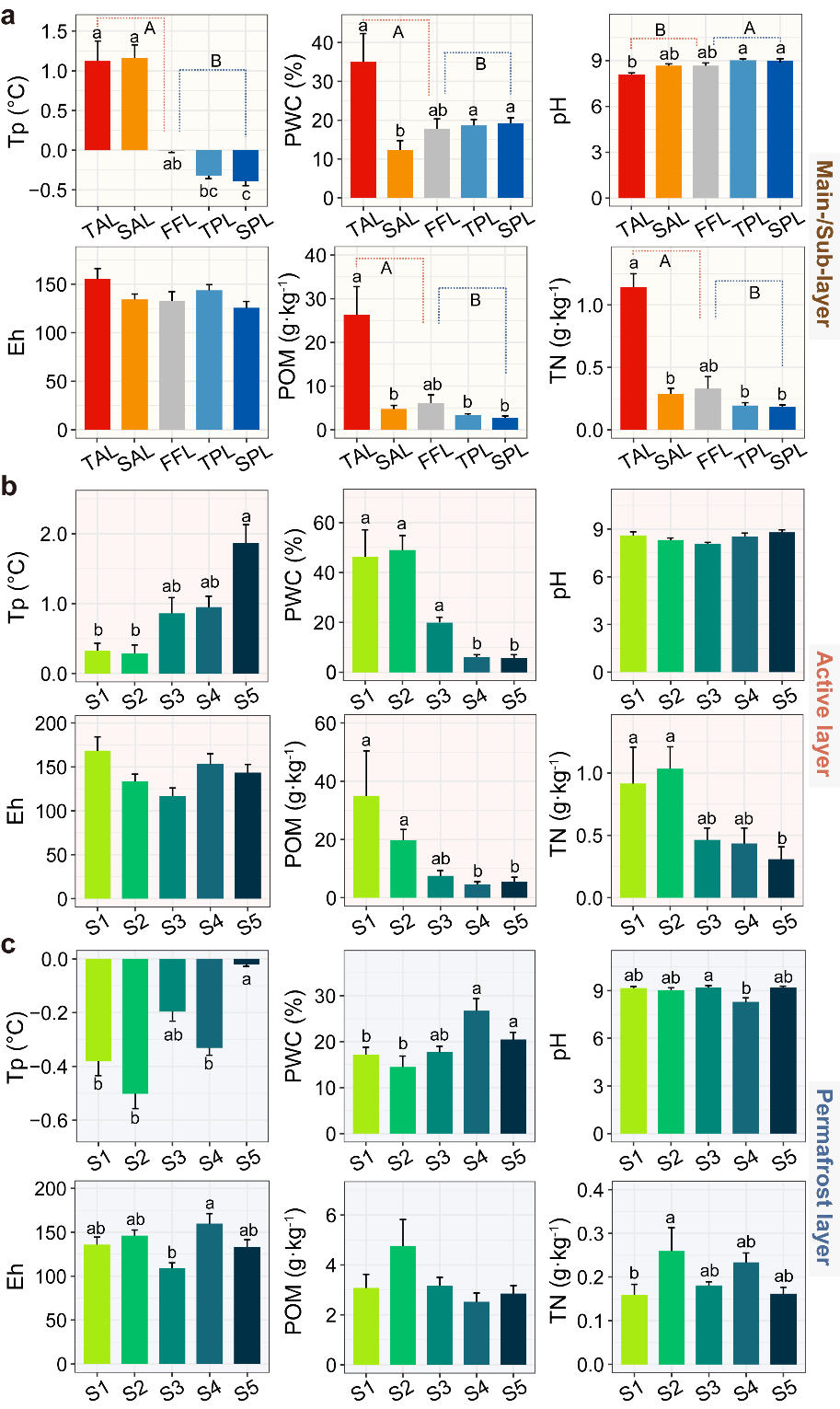


**Figure S1. Permafrost properties.** Variations in permafrost properties across the main- and sub-layers (a), as well as in the active (b) and permafrost layers (c) along the gradient of permafrost degradation. S1 to S5 characterizes a gradient of permafrost degradation. Sample sizes of main-layers are n = 51 and 74 for the active and permafrost layers, respectively. Sample sizes of sub-layers are n = 15, 31, 10, 49, and 20 for top-active layer (TAL), sub-active layer (SAL), frozen fringe layer (FFL), top-permafrost layer (TPL), and sub-permafrost layer (SPL), respectively. Sample sizes of sites in the active layer are n = 6, 7, 11, 12, and 15 for S1, S2, S3, S4, and S5, respectively. Sample sizes of sites in the permafrost layer are n = 19, 18, 14, 13, and 10 for S1, S2, S3, S4, and S5, respectively. Tp, permafrost temperature; PWC, permafrost water content; Eh, redox potential; POM, permafrost organic matter; TN, total nitrogen. TAL, SAL, and the upper part of FFL (bottom-active layer) belong to the active layer, the lower part of FFL (table-permafrost layer), TPL, and SPL belong to the permafrost layer. S1 represents the least degraded site, while S5 represents the most degraded site. Statistical significance is based on Kruskal-Wallis tests; Lowercase letters represent the significance of differences among sub-layers (TAL, SAL, FFL, TPL, and SPL) and different sites (from S1 to S5) in the active and permafrost layers, and uppercase letters represent the significance of differences between main-layers (active and permafrost layers).


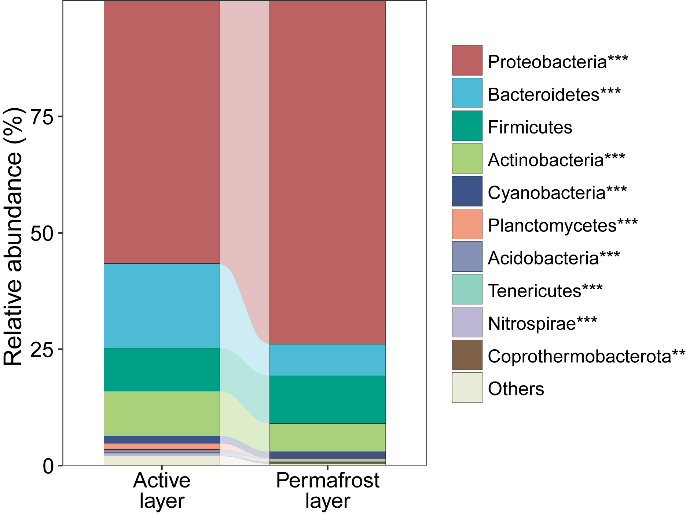


**Figure S2. Composition of bacterial communities in the active and permafrost layers.** Variations in relative abundances of the major phyla between the active and permafrost layers. Sample sizes are n = 51 and 74 for the active and permafrost layers, respectively. Statistical significance is based on Kruskal-Wallis tests. Asterisks indicate statistical significance (****p* < 0.001, ***p* < 0.01, and **p* < 0.05).
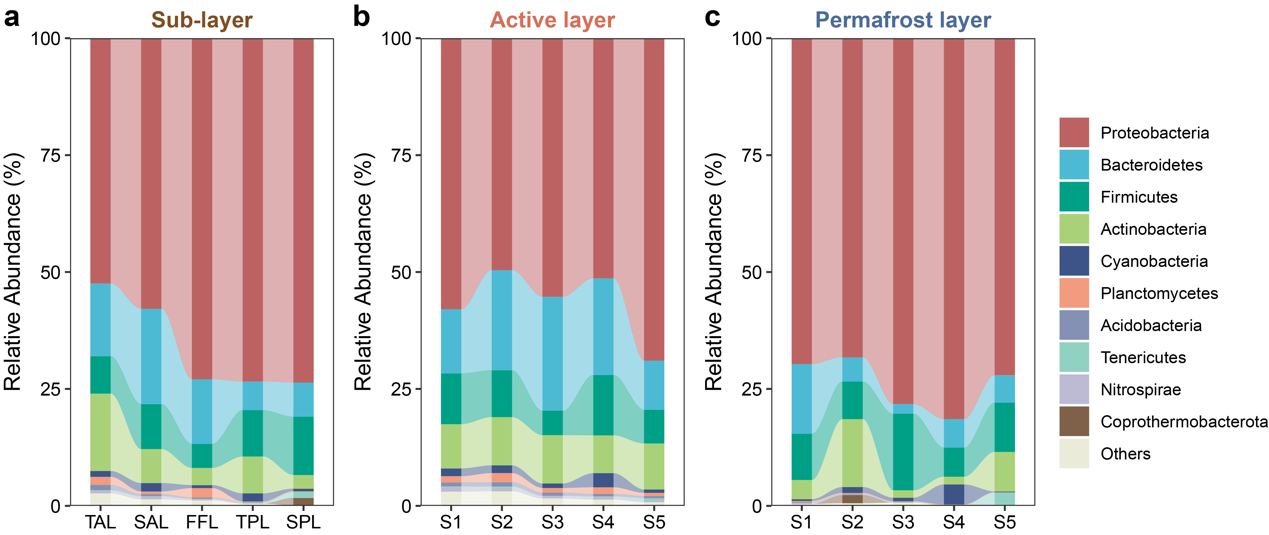


**Figure S3. Composition of bacterial communities across the sub-layers and along the gradient of permafrost degradation.** Variations in relative abundances of the major phyla across the sub-layers (a), as well as in the active (b) and permafrost layers (c) along the gradient of permafrost degradation. Sample sizes of sub-layers are n = 15, 31, 10, 49, and 20 for top-active layer (TAL), sub-active layer (SAL), frozen fringe layer (FFL), top-permafrost layer (TPL), and sub-permafrost layer (SPL), respectively. Sample sizes of sites in the active layer are n = 6, 7, 11, 12, and 15 for S1, S2, S3, S4, and S5, respectively. Sample sizes of sites in the permafrost layer are n = 19, 18, 14, 13, and 10 for S1, S2, S3, S4, and S5, respectively.


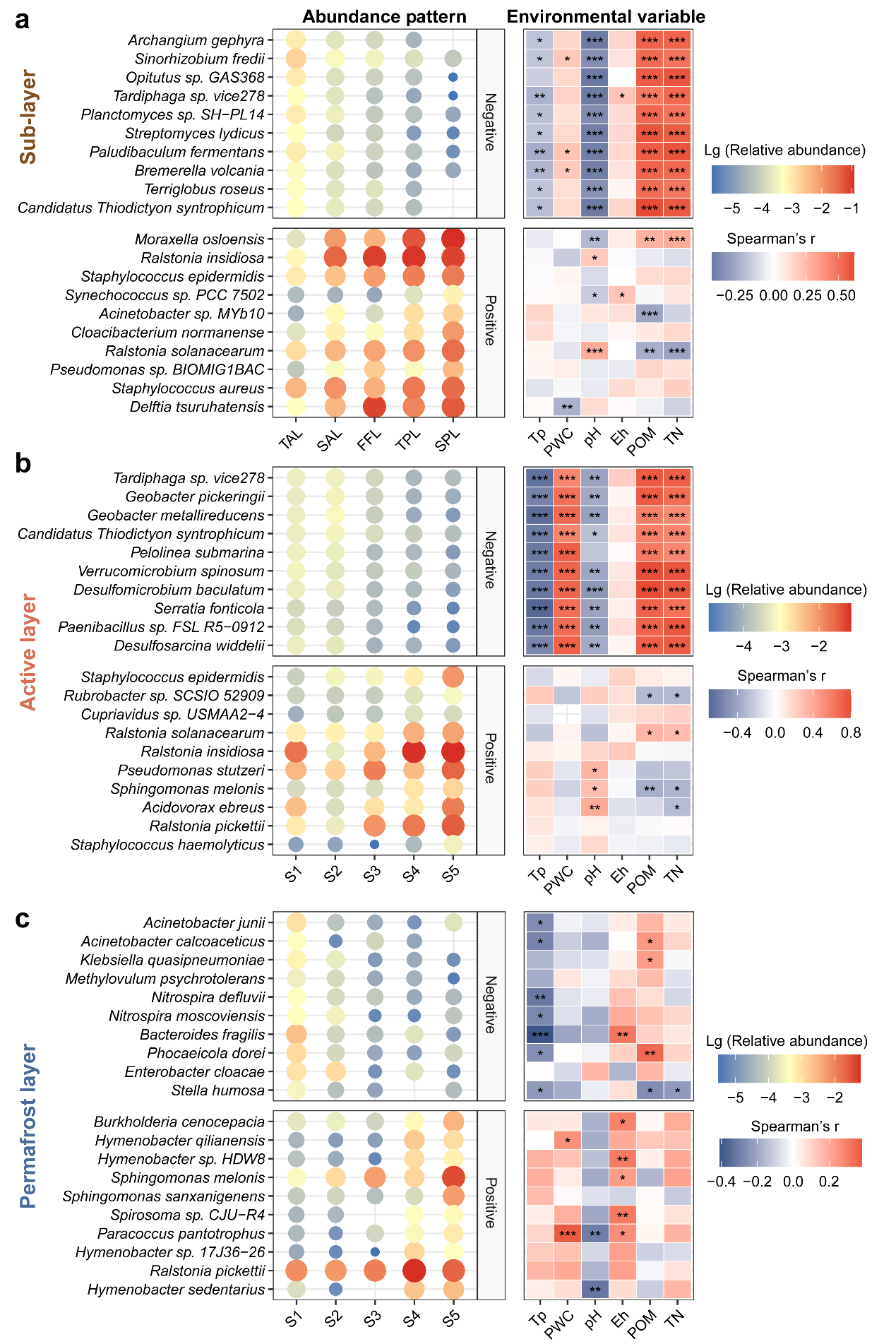


**Figure S4. Abundance pattern of bacterial species and their environment drivers.** The top 20 species exhibiting the strongest linear trends across the sub-layers (a), as well as in the active (b) and permafrost layers (c) along the gradient of permafrost degradation. Species are categorized into two groups, which includes those increasing (Positive) and decreasing (Negative) across the sub-layers/degradation gradient. In each panel, the left plot displays the variations in relative abundance, where bubble size and color intensity represent the standardized relative abundance. Sample sizes for both linear trend and Spearman correlation analyses are n = 150 for sub-layers, n = 51 for the active layer, and n = 79 for the permafrost layer. Asterisks indicate statistical significance (****p* < 0.001, ***p* < 0.01, and **p* < 0.05). TAL, top-active layer; SAL, sub-active layer; FFL, frozen fringe layer; TPL, top-permafrost layer; SPL, and sub-permafrost layer.


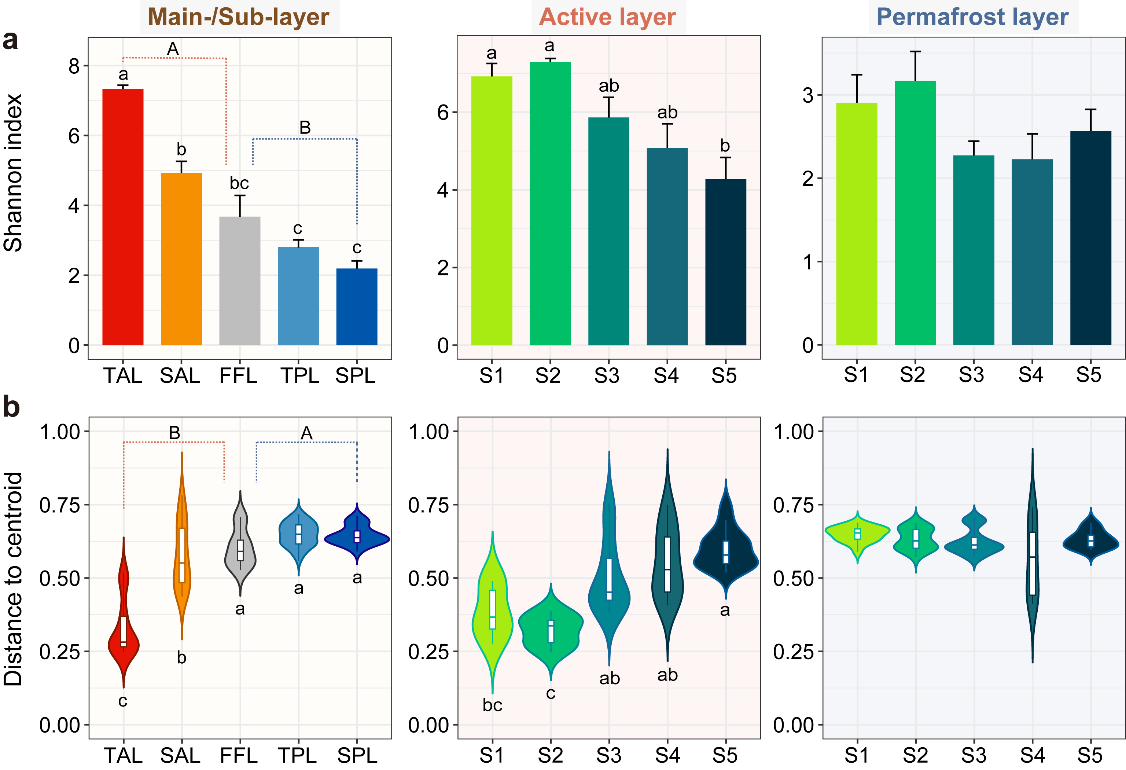


**Figure S****5. *α*- and *β*-diversity of bacterial communities.** (a) Variations in bacterial Shannon index across the main- and sub-layers, and the gradient of permafrost degradation. (b) Variations in distance of samples to group centroids across the main- and sub-layers, and the degradation gradient. Sample sizes of main-layers are n = 51 and 74 for the active and permafrost layers, respectively. Sample sizes of sub-layers are n = 15, 31, 10, 49, and 20 for the top-active layer (TAL), sub-active layer (SAL), frozen fringe layer (FFL), top-permafrost layer (TPL), and sub-permafrost layer (SPL), respectively. Sample sizes of sites in the active layer are n = 6, 7, 11, 12, and 15 for S1, S2, S3, S4, and S5, respectively. Sample sizes of sites in the permafrost layer are n = 19, 18, 14, 13, and 10 for S1, S2, S3, S4, and S5, respectively. S1 to S5 characterizes a gradient of permafrost degradation. Boxplots show median and interquartile range. Data are presented as mean ± s.e.m. Statistical significance is based on Kruskal-Wallis tests; Lowercase letters represent the significance of differences among sub-layers and different sites in the active and permafrost layers, and uppercase letters represent the significance of differences between main-layers.


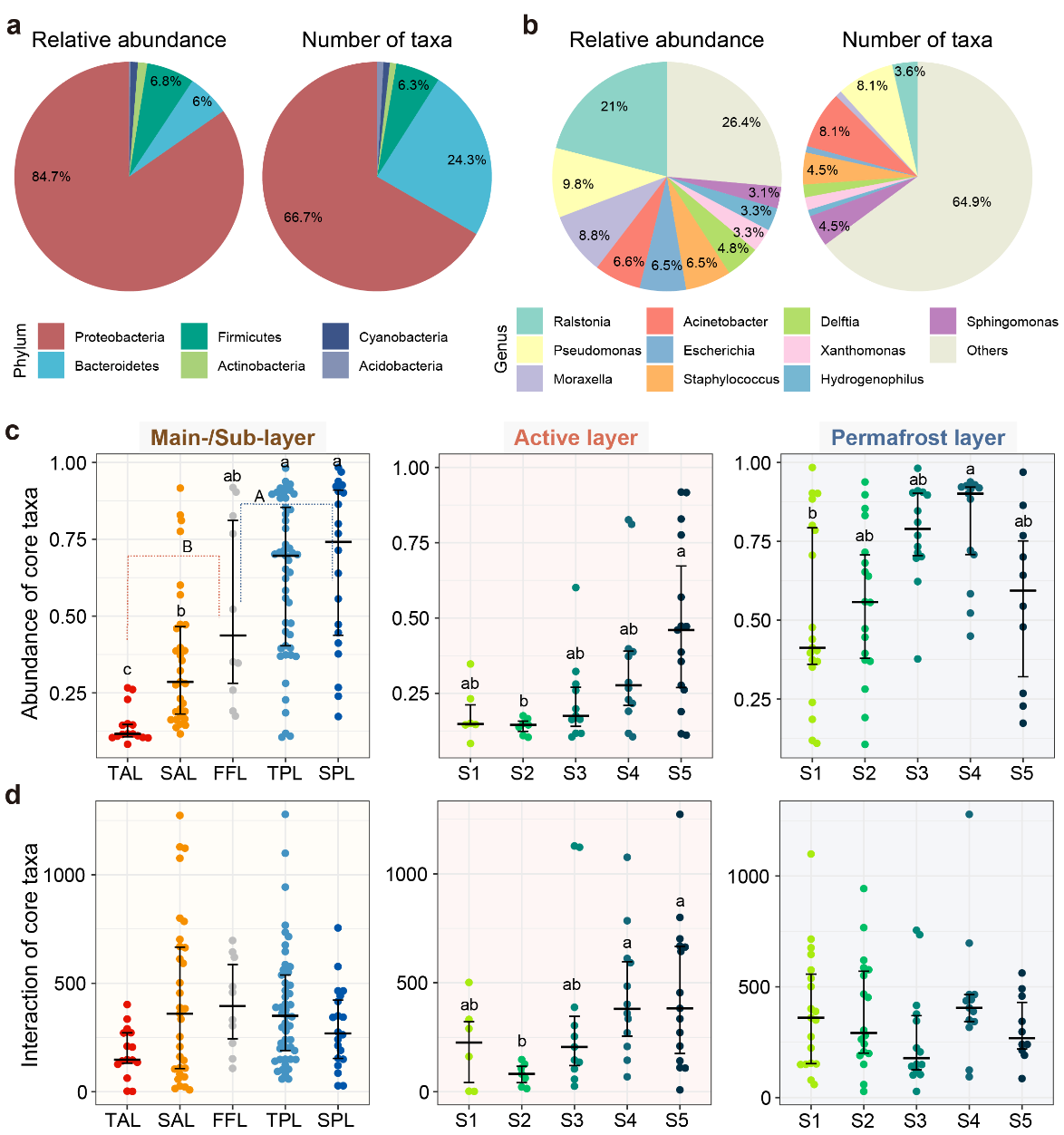


**Figure S6. Core taxa in bacterial communities.** (a) Taxonomic composition of core taxa at the phylum level including the number of taxa and relative abundance. (b) Taxonomic composition of core taxa at the genus level including the number of taxa and relative abundance. Only the top 10 genera in terms of relative abundance are displayed, while the rest were classified as “Others”. Only those with a proportion exceeding 2% display labels. (c) Variations in the relative abundance of core taxa across the main- and sub-layers, and the gradient of permafrost degradation. (d) Variations in the interaction of core taxa across the main- and sub-layers, and the degradation gradient. The interaction of core taxa is represented by the number of edges in the Single SparCC networks of core taxa. Sample sizes of main-layers are n = 51 and 74 for the active and permafrost layers, respectively. Sample sizes of sub-layers are n = 15, 31, 10, 49, and 20 for top-active layer (TAL), sub-active layer (SAL), frozen fringe layer (FFL), top-permafrost layer (TPL), and sub-permafrost layer (SPL), respectively. Sample sizes of sites in the active layer are n = 6, 7, 11, 12, and 15 for S1, S2, S3, S4, and S5, respectively. Sample sizes of sites in the permafrost layer are n = 19, 18, 14, 13, and 10 for S1, S2, S3, S4, and S5, respectively. S1 to S5 characterizes a gradient of permafrost degradation. Boxplots show median and interquartile range. Data are presented as mean ± s.e.m. Statistical significance is based on Kruskal-Wallis tests; Lowercase letters represent the significance of differences among sub-layers and different sites in the active and permafrost layers, and uppercase letters represent the significance of differences between main-layers.


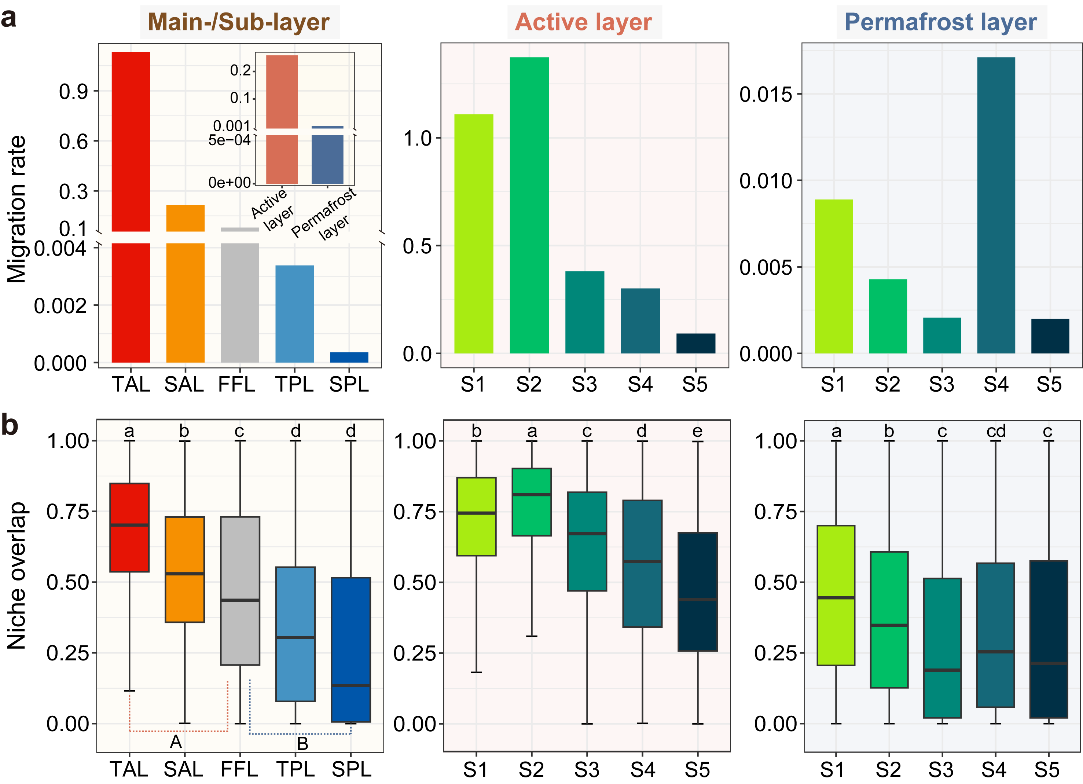


**Figure S7. Migration rates and niche overlaps of bacterial communities.** (a) Variations in migration rates across the main- and sub-layers, and the gradient of permafrost degradation. S1 to S5 characterizes a gradient of permafrost degradation. (b) Variations in niche overlaps across the main- and sub-layers, and the degradation gradient. Niche overlaps are calculated for each pair of bacterial species. Sample sizes of main-layers are n = 51 and 74 for the active and permafrost layers, respectively. Sample sizes of sub-layers are n = 15, 31, 10, 49, and 20 for top-active layer (TAL), sub-active layer (SAL), frozen fringe layer (FFL), top-permafrost layer (TPL), and sub-permafrost layer (SPL), respectively. Sample sizes of sites in the active layer are n = 6, 7, 11, 12, and 15 for S1, S2, S3, S4, and S5, respectively. Sample sizes of sites in the permafrost layer are n = 19, 18, 14, 13, and 10 for S1, S2, S3, S4, and S5, respectively. Boxplots show median and interquartile range. Data are presented as mean ± s.e.m. Statistical significance is based on Kruskal-Wallis tests; Lowercase letters represent the significance of differences among sub-layers and different sites in the active and permafrost layers, and uppercase letters represent the significance of differences between main-layers.


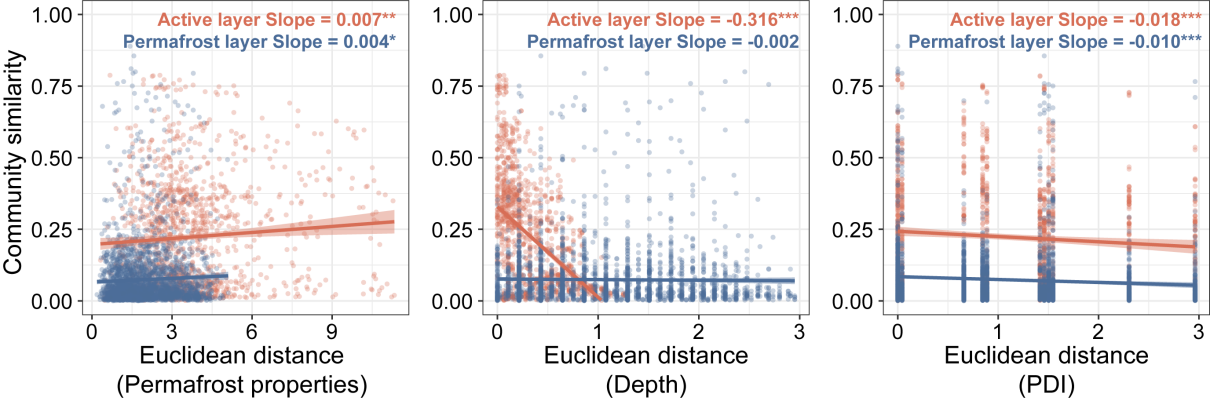


**Figure S8. Relationships between bacterial community similarity and environmental distance.** Differences of relationships between community similarity (1 - the Bray-Curtis distance) and environmental distance (Euclidean distances based on permafrost properties, depth, and PDI, respectively) between the active (*n* = 1275 pairwise distances) and permafrost layers (*n* = 2701 pairwise distances). PDI, permafrost degradation index. Asterisks indicate statistical significance (****p* < 0.001, ***p* < 0.01, and **p* < 0.05).


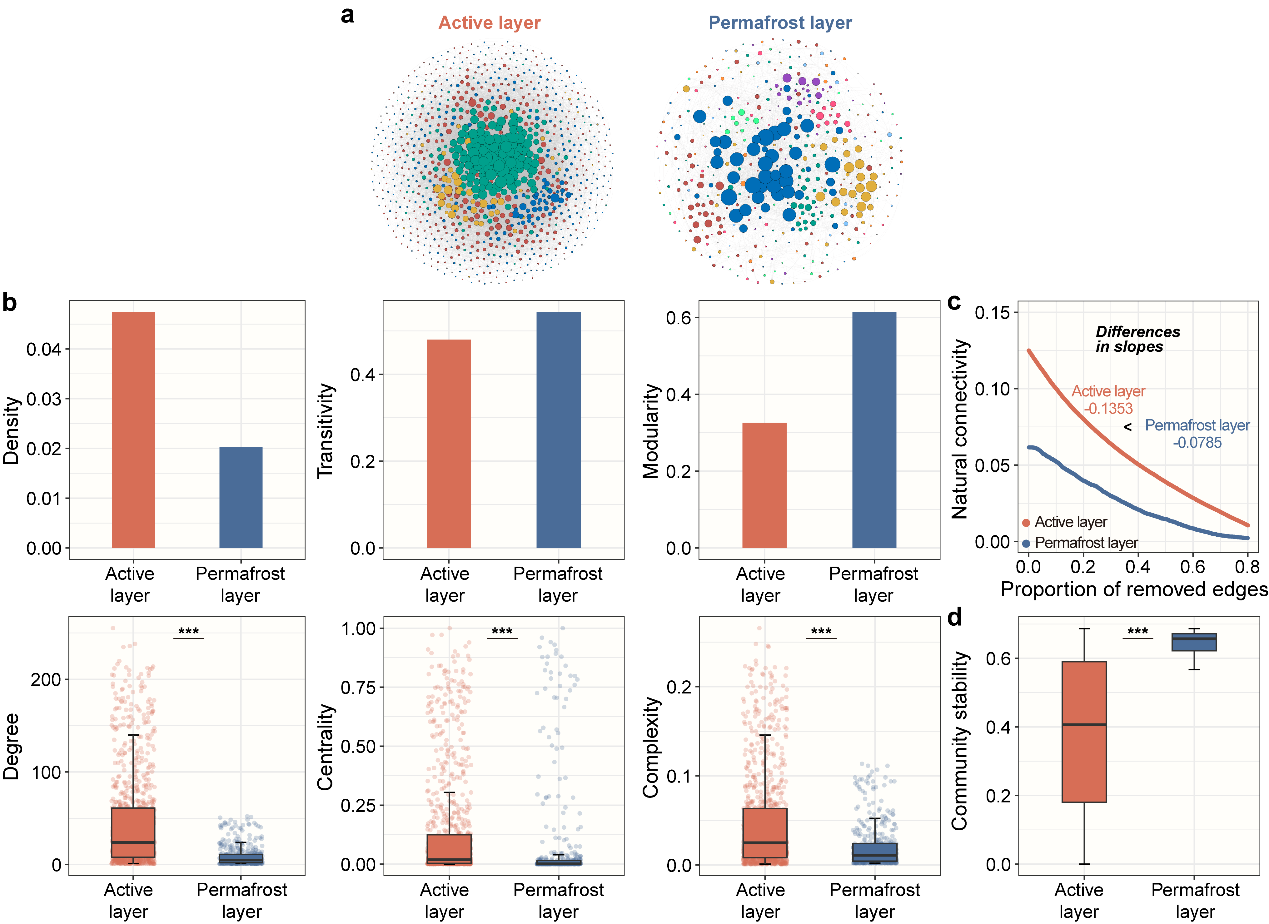


**Figure S9. Co-occurrence networks and stability of bacterial communities across the main-layers.** (a) Nodes represent individual species whose size is positively correlated with the node degree; multiple node colors represent different modules. The sample size is *n* = 25 per layer. (b) Variations in network topological properties including density, transitivity, modularity, degree, centrality (eigenvector), and complexity (linkage density; degree/node) between the active and permafrost layers. (c) Robustness analysis exhibits the relationships between natural connectivity and the proportion of removed edges; larger changes in natural connectivity upon the same proportion of removed edges indicate less network robustness. (d) Variations in community stability calculated by the average variation degree method between the active and permafrost layers. Sample sizes are *n* = 51 and 74 for the active and permafrost layers, respectively. Boxplots show median and interquartile range. Data are presented as mean ± s.e.m. Statistical significance is based on Kruskal-Wallis tests. Statistical significance is based on Kruskal-Wallis tests. Asterisks indicate statistical significance (****p* < 0.001, ***p* < 0.01, and **p* < 0.05).


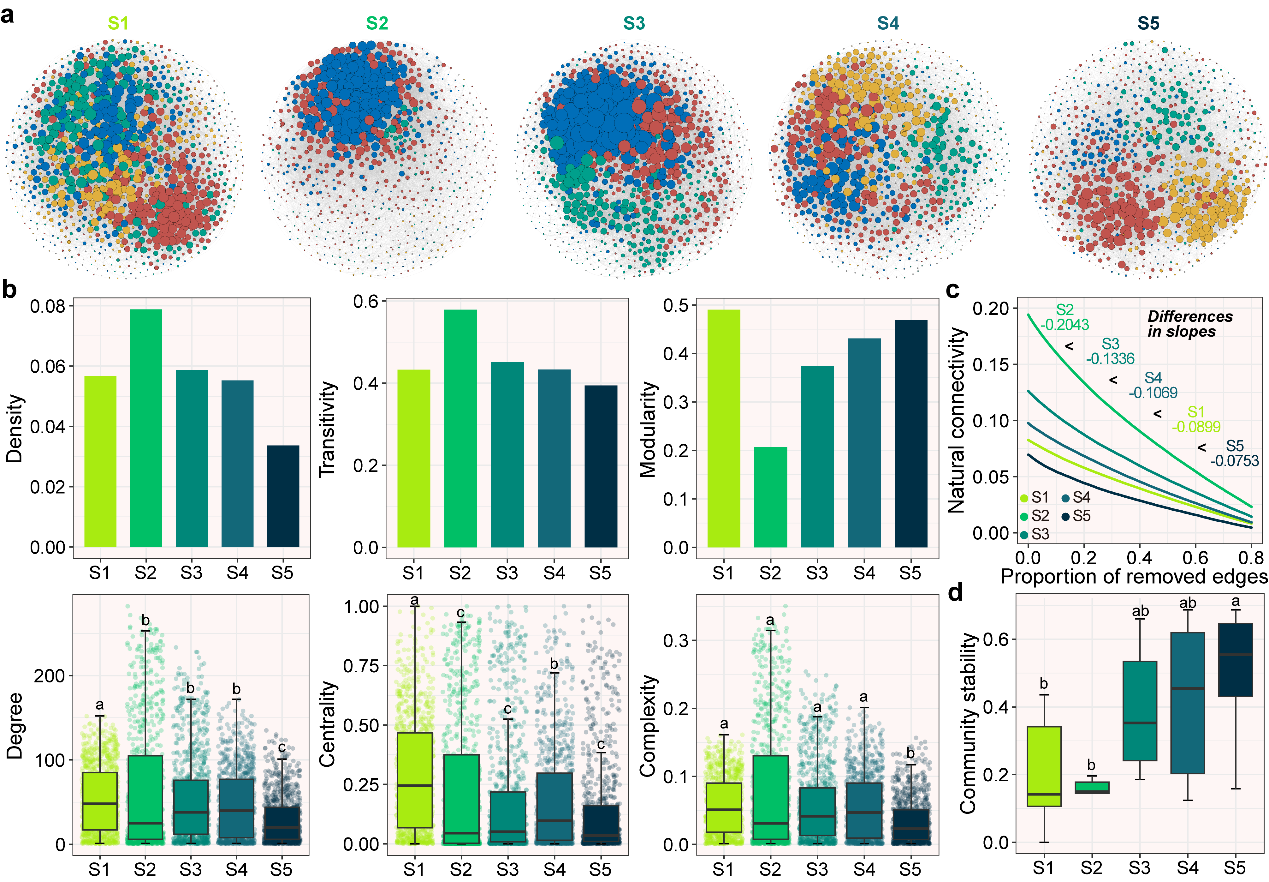


**Figure S10.** **Co-occurrence networks and stability of bacterial communities in the active layer along the gradient of permafrost degradation.** (a) Nodes represent individual species whose size is positively correlated with the node degree; multiple node colors represent different modules. (b) Variations in network topological properties including density, transitivity, modularity, degree, centrality (eigenvector), and complexity (linkage density; degree/node) in the active layer along the degradation gradient. (c) Robustness analysis exhibits the relationships between natural connectivity and the proportion of removed edges; larger changes in natural connectivity upon the same proportion of removed edges indicate less network robustness. (d) Variations in community stability calculated by the average variation degree method in the active layer along the degradation gradient. S1 to S5 characterizes a gradient of permafrost degradation. Sample sizes are n = 6, 7, 11, 12, and 15 for S1, S2, S3, S4, and S5, respectively. Boxplots show median and interquartile range. Data are presented as mean ± s.e.m. Statistical significance is based on Kruskal-Wallis tests.


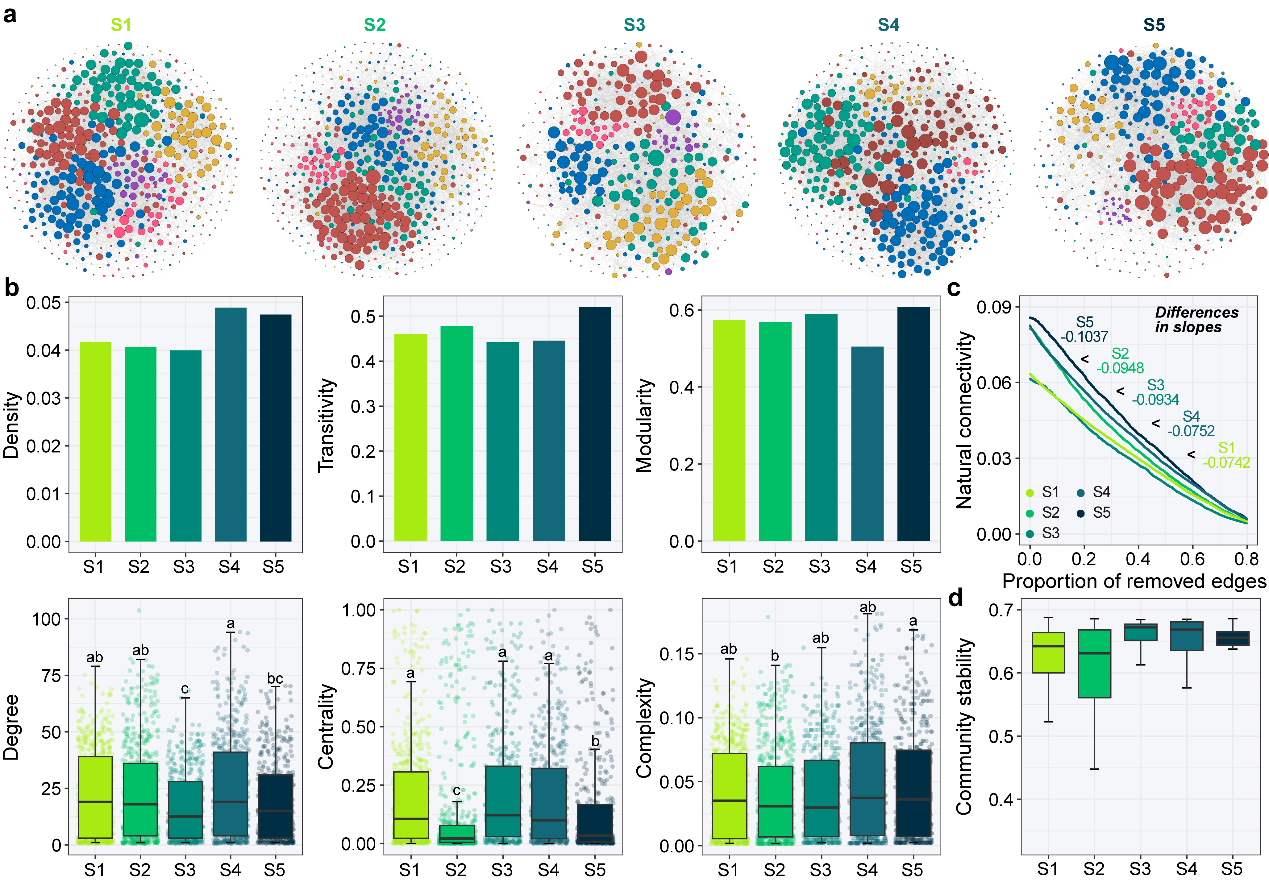


**Figure S11. Co-occurrence networks and stability of bacterial community in the permafrost layer along the gradient of permafrost degradation.** (a) Nodes represent individual species whose size is positively correlated with the node degree; multiple node colors represent different modules. (b) Variations in network topological properties including density, transitivity, modularity, degree, centrality (eigenvector), and complexity (linkage density; degree/node) in the permafrost layer along the degradation gradient. (c) Robustness analysis exhibits the relationships between natural connectivity and the proportion of removed edges; larger changes in natural connectivity upon the same proportion of removed edges indicate less network robustness. (d) Variations in community stability calculated by the average variation degree method in the permafrost layer along the degradation gradient. S1 to S5 characterizes a gradient of permafrost degradation. Sample sizes are n = 19, 18, 14, 13, and 10 for S1, S2, S3, S4, and S5, respectively. Boxplots show median and interquartile range. Data are presented as mean ± s.e.m. Statistical significance is based on Kruskal-Wallis tests.


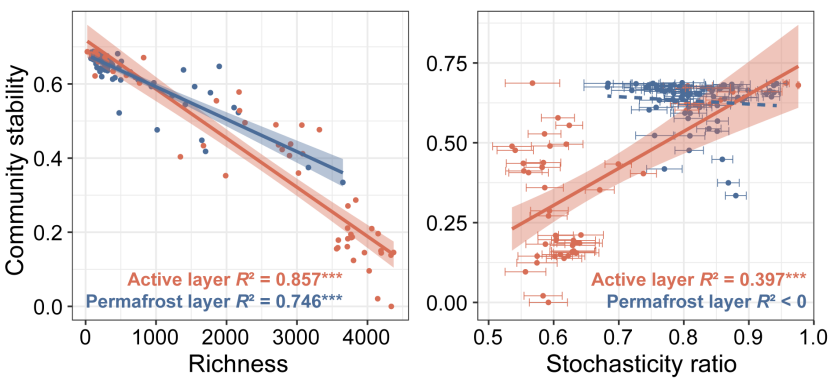


**Figure S12. Relationships of bacterial richness and stochasticity ratio with community stability.** Relationships of bacterial richness and stochasticity ratio with community stability in the active and permafrost layers. Asterisks indicate statistical significance (****p* < 0.001, ***p* < 0.01, and **p* < 0.05).


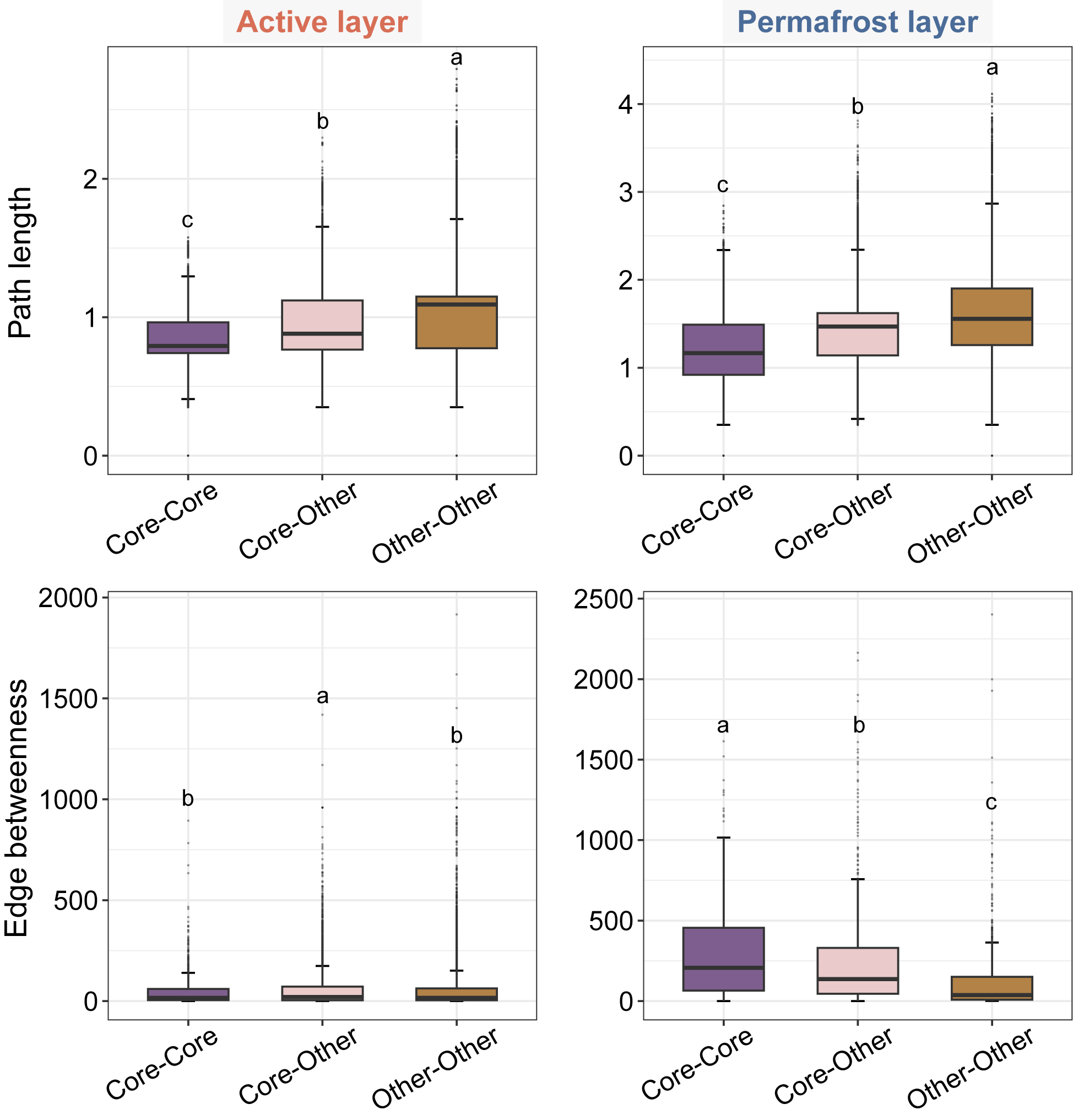


**Figure S13. Path length and edge betweenness for different types of edges in bacterial networks.** Variations in path length and edge betweenness among three types of edges in bacterial networks between the active and permafrost layers. Core-Core, Core-other, and Other-Other denote edges between any two nodes of core taxa, between any one node of core taxa and any one node of other taxa, and between any two nodes of other taxa, respectively. Boxplots show median and interquartile range. Data are presented as mean ± s.e.m. Statistical significance is based on Kruskal-Wallis tests.

**
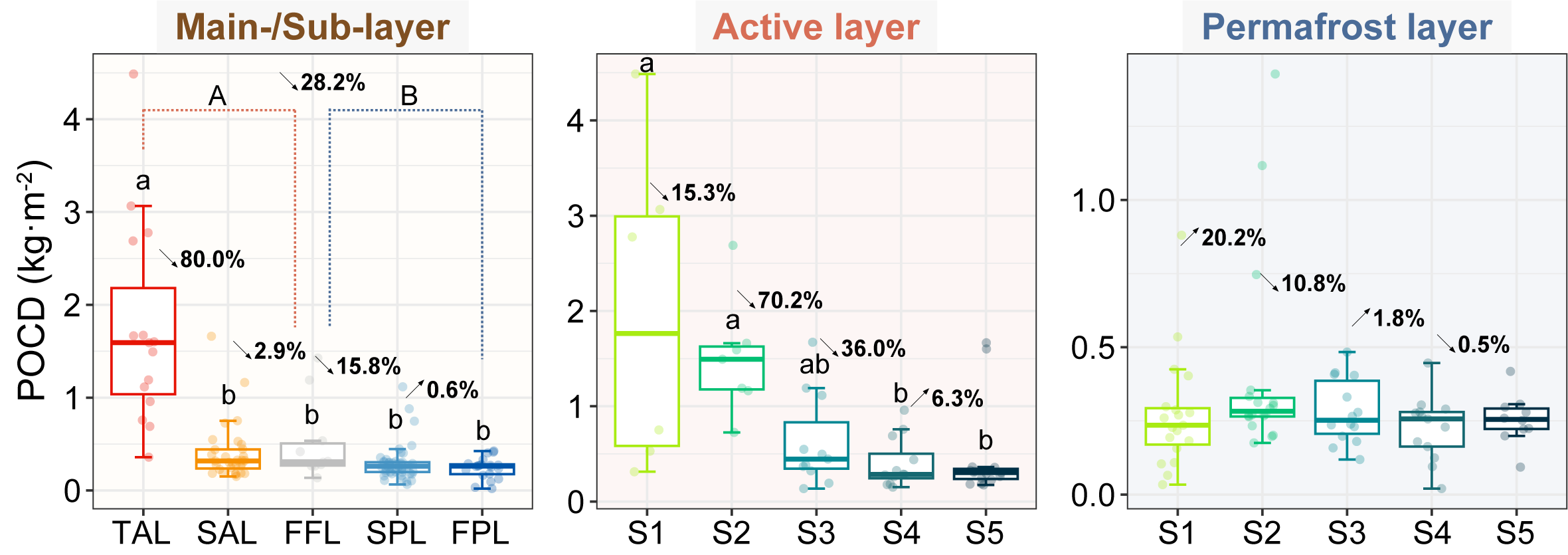
**

**Figure S14. Permafrost organic carbon density.** Variations in POCD across the main- and sub-layers and gradient of permafrost degradation in the active and permafrost layers. Sample sizes of main-layers are n = 51 and 74 for the active and permafrost layers, respectively. Sample sizes of sub-layers are n = 15, 31, 10, 49, and 20 for the top-active layer (TAL), sub-active layer (SAL), frozen fringe layer (FFL), top-permafrost layer (TPL), and sub-permafrost layer (SPL), respectively. Sample sizes of sites in the active layer are n = 6, 7, 11, 12, and 15 for S1, S2, S3, S4, and S5, respectively. Sample sizes of sites in the permafrost layer are n = 19, 18, 14, 13, and 10 for S1, S2, S3, S4, and S5, respectively. S1 to S5 characterizes a gradient of permafrost degradation. Boxplots show median and interquartile range. Data are presented as mean ± s.e.m. Statistical significance is based on Kruskal-Wallis tests; Lowercase letters represent the significance of differences among sub-layers and different sites in the active and permafrost layers, and uppercase letters represent the significance of differences between main-layers.

**
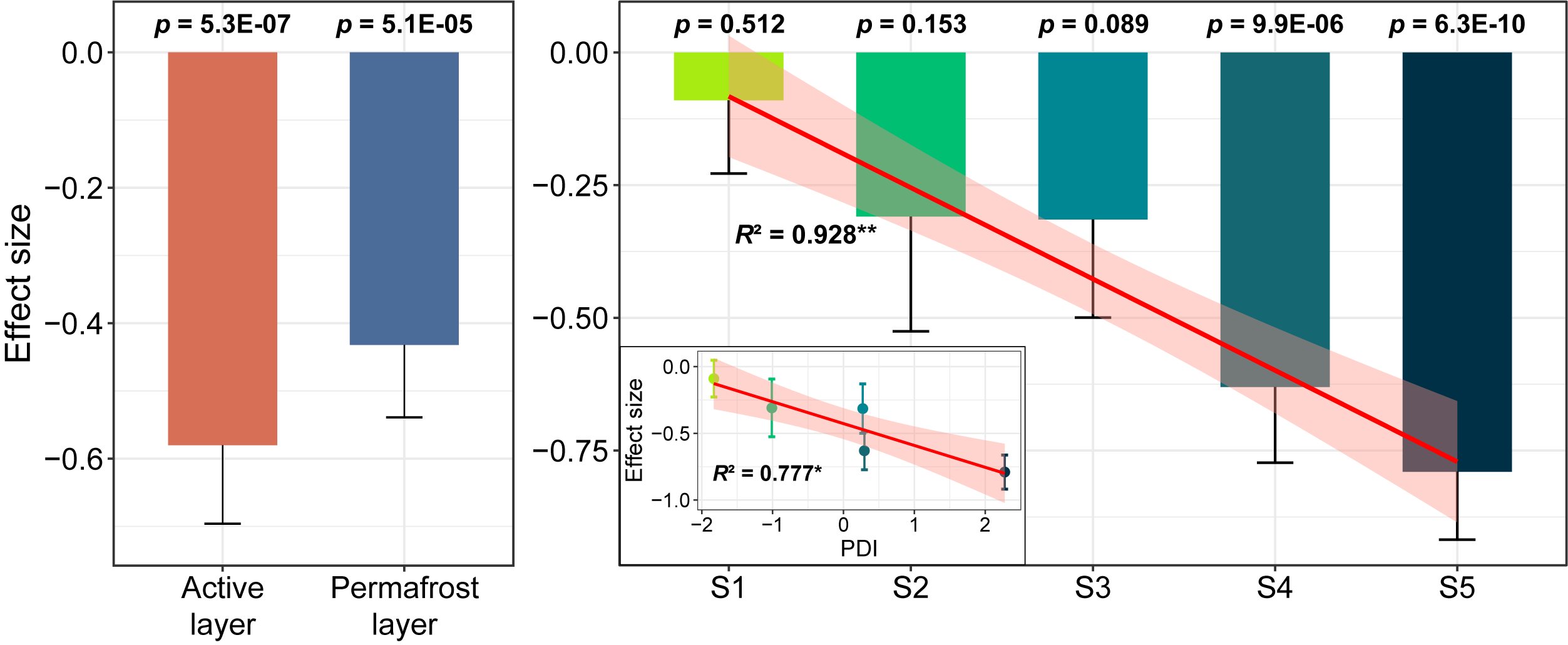
**

**Figure S15. Effects of bacterial community stability on permafrost C storage.** The estimated effect sizes are regression coefficients based on rescaled response variables (with zero mean and unit standard deviation) in the linear mixed-effects models. The relationship of this effect with PDI is displayed in the lower left corner. PDI, permafrost degradation index. Boxplots show median and interquartile range. Data are presented as mean ± s.e.m. Statistical significance of the estimated effects is based on Wald type II *χ*² tests. Asterisks indicate statistical significance (****p* < 0.001, ***p* < 0.01, and **p* < 0.05).


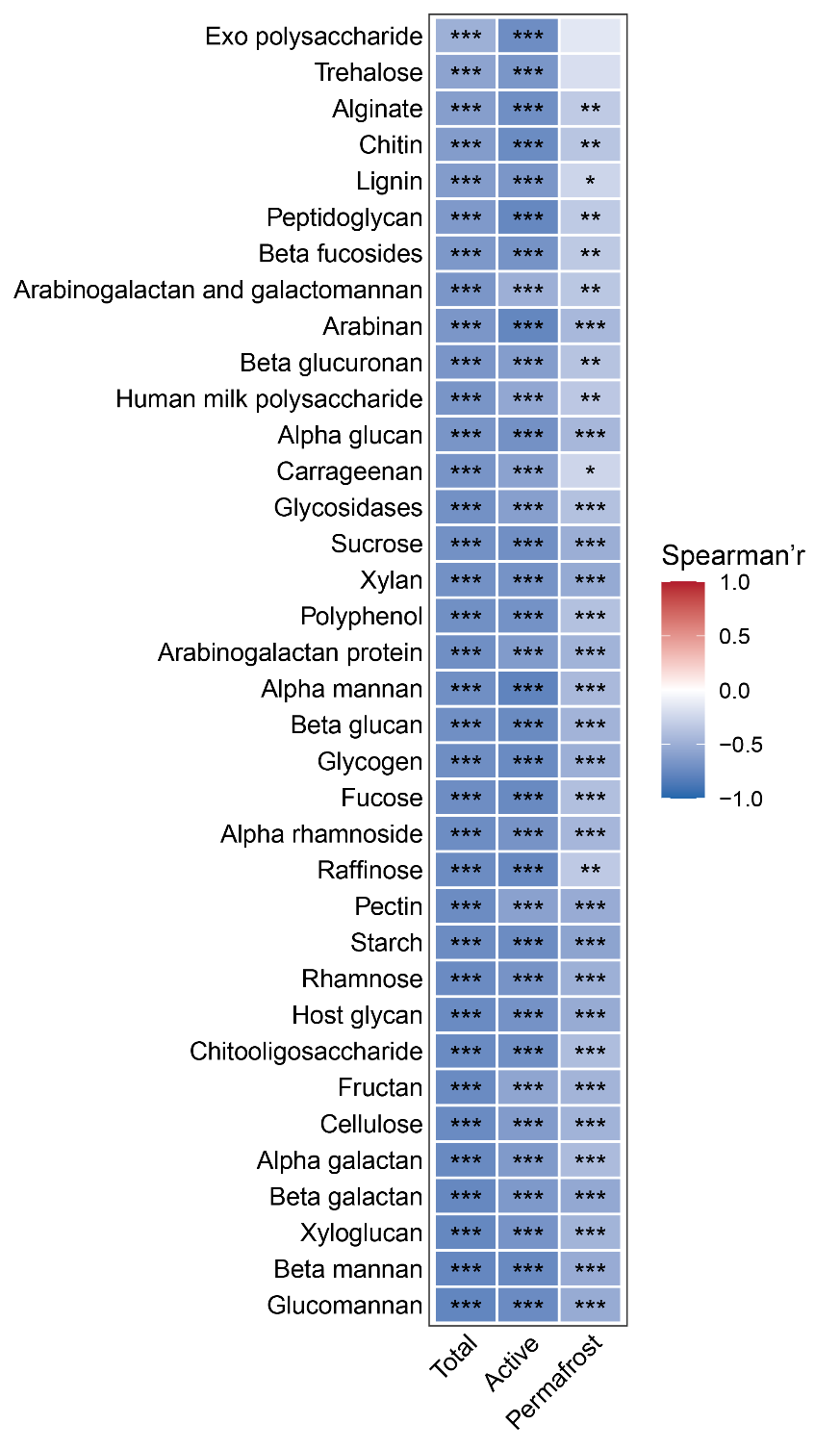


**Figure S16. The relationships between carbohydrate-active enZymes (CAZymes) and bacterial community stability.** The Spearman’s correlations between CAZymes and bacterial community stability in the total, active, and permafrost layers (****p* < 0.001, ***p* < 0.01, and **p* < 0.05). Sample sizes are n = 150, 51, 74 for the total, active, and permafrost layer, respectively.


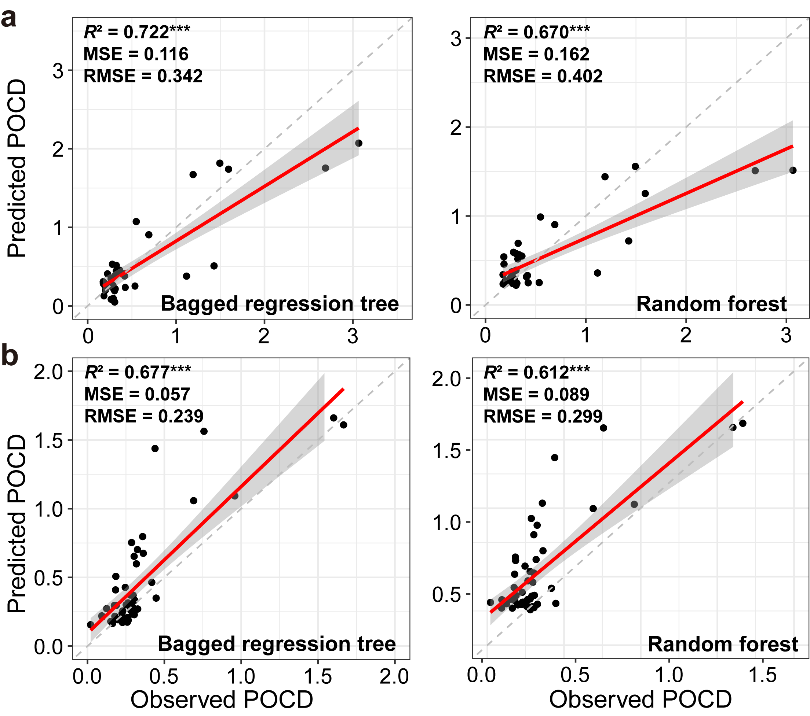


**Figure S17. Prediction of permafrost organic carbon density by bacterial communities using machine learning.** (a) Relationship between observed POCD and predicted POCD in test dataset (30% of the dataset) derived from bagged regression tree and random forest models (n = 38 samples), respectively. (b) Models trained on the dataset from S1, S2, and S3 to predict POCD in S4 and S5 using bagged regression tree and random forest models (n = 50 samples), respectively. The grey diagonal lines show the theoretical curve for perfect predictions. POCD, permafrost organic carbon density; MSE, mean squared error; RMSE, root mean squared error. Asterisks indicate statistical significance (****p* < 0.001, ***p* < 0.01, and **p* < 0.05).


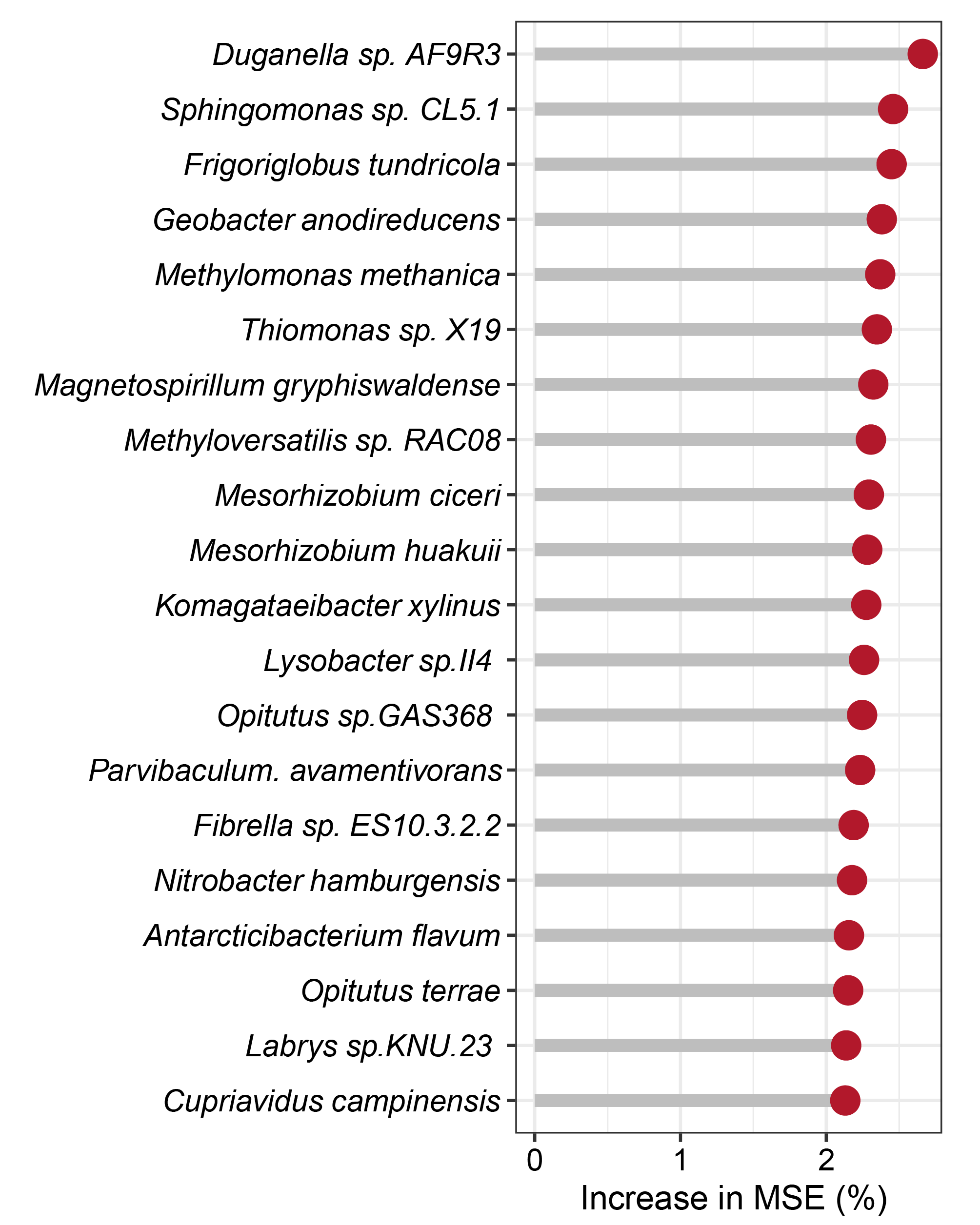


**Figure S18. The top 20 most important bacterial predictors of POCD.** Importance of top 20 species-level taxa ranked by increase in mean squared error (MSE). POCD, permafrost organic carbon density.

**
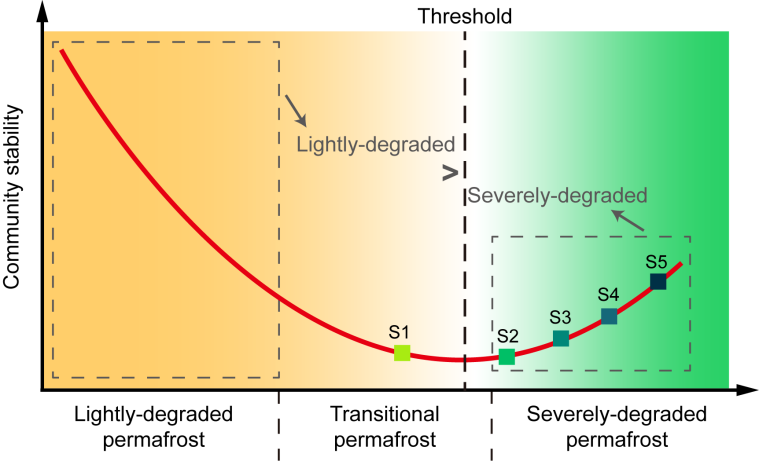
**

**Figure S19. A hypothesis about the variation of bacterial community stability in the active layer during the processes of permafrost degradation.** Based on the classification of permafrost degradation stages, the five sampling sites belonged to transitional (S1) and severely-degraded permafrost (from S2 to S5). A hypothesis is proposed that community stability decreases during the lightly-degraded stage, reaches a turning point during the transitional stage, and then increases slightly during the severely-degraded stage, with an overall higher level in the lightly-degraded stage than the severely-degraded stage.

**
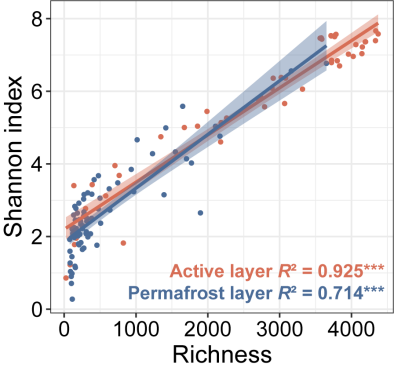
**

**Figure S20. Relationships of bacterial richness with the Shannon index.** Since bacterial richness is highly correlated with the Shannon index in both the active and permafrost layers, it is used to represent bacterial *α*-diversity in subsequent analyses. Asterisks indicate the statistical significance (****p* < 0.001, ***p* < 0.01, and **p* < 0.05).


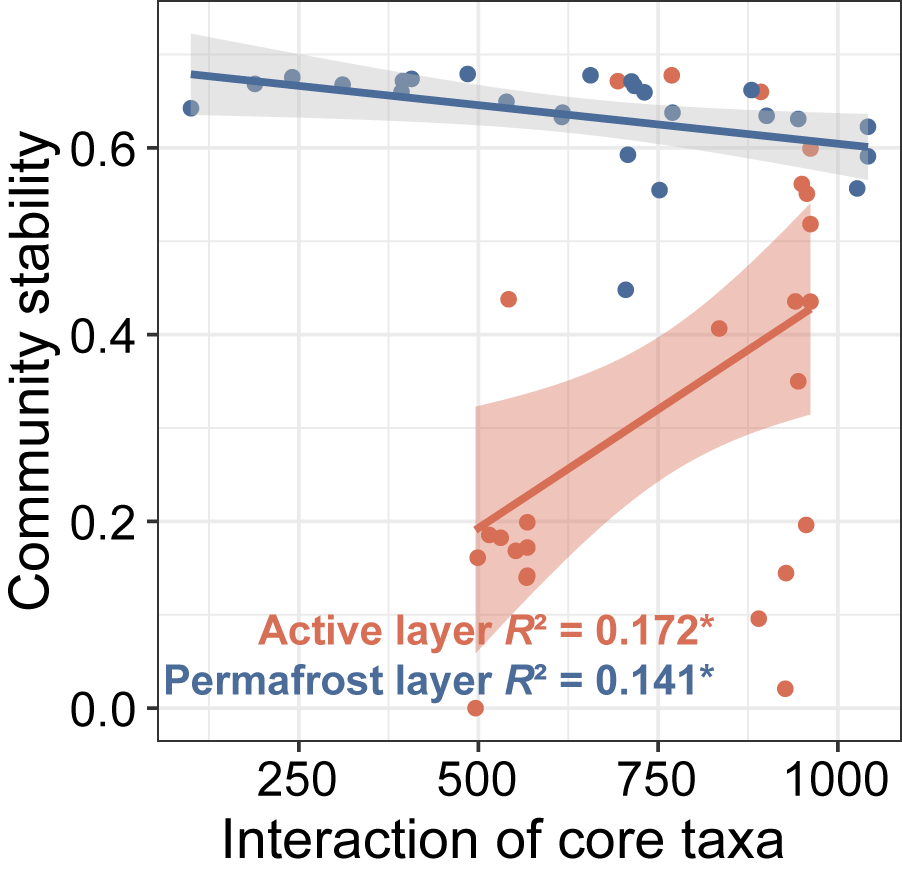


**Figure S21. Relationships of the interaction of bacterial core taxa with community stability.** The interaction of core taxa extracted from SparCC networks is fitted to the averaged data of community stability (n = 25 samples per layer) to demonstrate the feasibility of the Single SparCC network analysis and the accuracy of results. Asterisks indicate statistical significance (****p* < 0.001; ***p* < 0.01; and **p* < 0.05).

**
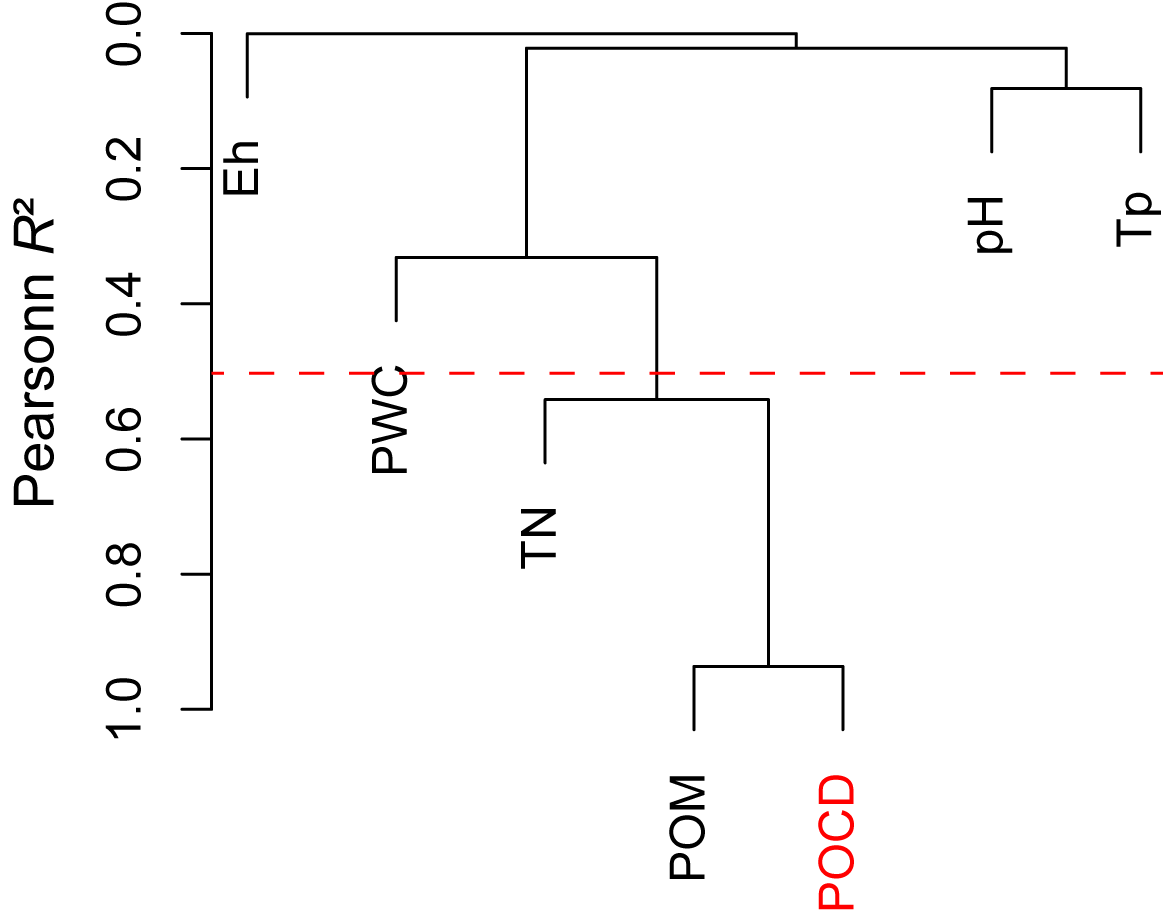
**

**Figure S22. Cluster analysis of the measured permafrost properties.** POM and TN were removed from permafrost properties before performing structural equation modelling due to their high Pearson correlations (*R*^2^ > 0.5) with POCD. Tp, permafrost temperature; PWC, permafrost water content; Eh, redox potential; POM, permafrost organic matter; POCD, permafrost organic carbon density.

**Table S1. Location, elevation, vegetation type, and active layer thickness of sampling sites.**

| Site | Longitude/ latitude (E/N) | Elevation (m) | Vegetation type | Active layer thickness (m) |
| --- | --- | --- | --- | --- |
| S1 | 98°46′02″/ 38°23′44″ | 4090 | Alpine swamp meadow | 1.2 |
| S2 | 98°13′45″/ 38°19′58″ | 3945 | Alpine meadow | 1.5 |
| S3 | 98°13′45″/ 38°19′56″ | 3967 | Alpine meadow | 3.5 |
| S4 | 98°12′20″/ 38°33′02″ | 3832 | Alpine steppe | 4.0 |
| S5 | 98°19′25″/ 38°28′33″ | 3890 | Alpine meadow | 6.0 |

**Table S2. Relative abundances** **of the major phyla in bacterial communities.**

|  | Sub-layer | | | | | |
| --- | --- | --- | --- | --- | --- | --- |
| Phylum | | TAL | SAL | FFL | TPL | SPL |
| Proteobacteria | | 52.433±3.004 b | 57.857±4.245 ab | 72.930±7.599 ab | 73.418±3.157 ab | 73.627±5.746 a |
| Bacteroidetes | | 15.552±4.296 ab | 20.381±3.431 a | 13.812±6.448 ab | 6.127±1.457 b | 7.313±3.034 b |
| Firmicutes | | 8.014±0.935 | 9.615±2.570 | 5.146±1.784 | 9.897±1.704 | 12.451±3.983 |
| Actinobacteria | | 16.557±2.203 a | 7.269±1.044 ab | 3.682±1.547 b | 7.895±2.191 bc | 2.941±0.928 c |
| Cyanobacteria | | 1.238±0.199 a | 1.807±0.515 ab | 0.627±0.294 b | 1.719±0.851 c | 0.538±0.199 c |
| Planctomycetes | | 1.717±0.195 a | 0.545±0.091 b | 2.022±1.003 a | 0.159±0.062 b | 0.118±0.073 b |
| Acidobacteria | | 1.128±0.106 a | 0.439±0.067 ab | 0.255±0.130 bc | 0.099±0.033 c | 0.030±0.019 c |
| Tenericutes | | 0.137±0.036 a | 0.093±0.015 a | 0.049±0.044 b | 0.114±0.069 b | 1.237±1.098 ab |
| Nitrospirae | | 0.521±0.098 a | 0.563±0.113 a | 0.262±0.145 ab | 0.097±0.045 b | 0.042±0.032 b |
| Coprothermobacterota | | 0.005±0.002 a | 0.005±0.002 ab | 0.009±0.008 b | 0.002±0.001 b | 1.584±1.582 ab |
|  | Active layer | | | | | |
|  | | S1 | S2 | S3 | S4 | S5 |
| Proteobacteria | | 57.935±4.165 | 49.594±4.072 | 55.280±6.918 | 51.339±7.587 | 68.928±5.222 |
| Bacteroidetes | | 13.735±4.014 | 21.403±4.809 | 24.381±7.031 | 20.683±6.296 | 10.508±3.326 |
| Firmicutes | | 10.862±1.975 | 10.017±1.436 | 5.228±0.780 | 12.901±6.144 | 7.230±2.066 |
| Actinobacteria | | 9.472±2.637 | 10.337±3.023 | 10.342±2.683 | 8.091±2.361 | 9.843±2.224 |
| Cyanobacteria | | 1.653±0.301 | 1.640±0.314 | 0.945±0.213 | 3.016±1.260 | 0.699±0.164 |
| Planctomycetes | | 1.338±0.416 | 1.954±0.739 | 0.977±0.261 | 1.403±0.707 | 0.674±0.253 |
| Acidobacteria | | 0.841±0.117 | 0.917±0.110 | 0.681±0.178 | 0.523±0.138 | 0.487±0.149 |
| Tenericutes | | 0.136±0.034 | 0.212±0.239 | 0.091±0.026 | 0.109±0.042 | 0.535±0.016 |
| Nitrospirae | | 0.938±0.204 | 0.759±0.182 | 0.478±0.127 | 0.559±0.223 | 0.294±0.106 |
| Coprothermobacterota | | 0.012±0.004 | 0.005±0.002 | 0.002±0.002 | 0.006±0.005 | 0.001±0.001 |
|  | Permafrost layer | | | | | |
|  | | S1 | S2 | S3 | S4 | S5 |
| Proteobacteria | | 69.679±5.451 | 68.234±5.648 | 78.222±6.271 | 81.439±5.385 | 72.065±7.763 |
| Bacteroidetes | | 14.890±4.880 | 5.190±1.660 | 2.086±0.713 | 6.087±2.483 | 5.881±2.660 |
| Firmicutes | | 9.897±2.522 | 8.024±2.030 | 16.358±5.911 | 6.229±2.888 | 10.522±3.871 |
| Actinobacteria | | 4.120±0.949 ab | 14.534±5.268 a | 1.590±0.620 b | 1.674±0.571 ab | 8.462±3.795 ab |
| Cyanobacteria | | 0.364±0.152 | 1.260±0.628 | 0.763±0.657 | 4.282±2.979 | 0.144±0.122 |
| Planctomycetes | | 0.232±0.134 | 0.187±0.092 | 0.120±0.078 | 0.137±0.107 | 0.041±0.034 |
| Acidobacteria | | 0.152±0.708 | 0.122±0.053 | 0.040±0.280 | 0.014±0.006 | 0.026±0.017 |
| Tenericutes | | 0.019±0.010 | 0.042±0.017 | 0.114±0.059 | 0.017±0.013 | 2.739±2.171 |
| Nitrospirae | | 0.213±0.108 | 0.084±0.045 | 0.025±0.018 | 0.005±0.003 | 0.021±0.020 |
| Coprothermobacterota | | 0.009±0.005 | 1.758±1.758 | 0.002±0.002 | 0.000±0.000 | 0.000±0.000 |

Variations in the relative abundances of major phyla across the main- and sub-layers, and the gradient of permafrost degradation. S1 to S5 characterizes a gradient of permafrost degradation. Statistical significance is based on Kruskal-Wallis tests. Sample sizes of sites in the active layer are n = 6, 7, 11, 12, and 15 for S1, S2, S3, S4, and S5, respectively. Sample sizes of sites in the permafrost layer are n = 19, 18, 14, 13, and 10 for S1, S2, S3, S4, and S5, respectively. TAL, top-active layer; SAL, sub-active layer; FFL, frozen fringe layer; TPL, top-permafrost layer; SPL, and sub-permafrost layer.

**Table S4. Affecting variables of bacterial composition and stochasticity ratio.**

|  | Active layer | | | | | Permafrost layer | | | | Active layer | Permafrost layer |
| --- | --- | --- | --- | --- | --- | --- | --- | --- | --- | --- | --- |
| X: variable | | Y~X | Y~lnX | lnY~X | lnY~lnX | Y~X | Y~lnX | lnY~X | lnY~lnX | Best model results | |
|  | | Composition | | | | | | | | | |
| Depth | | 0.481*** | 0.412*** | 0.455*** | 0.411*** | 0.014 | 0.014 | 0.020 | 0.000 | 0.481*** | 0.014 |
| PDI | | 0.083** | 0.028 | 0.086** | 0.026 | 0.077*** | 0.079*** | 0.062*** | 0.071*** | 0.086** | 0.079*** |
| Tp | | 0.113*** | 0.113*** | 0.104*** | 0.104*** | 0.116*** | 0.081*** | 0.103*** | 0.073*** | 0.113*** | 0.116*** |
| PWC | | 0.169*** | 0.169*** | 0.160*** | 0.160*** | 0.000 | 0.000 | 0.014 | 0.014 | 0.169*** | 0.014 |
| pH | | 0.224*** | 0.170*** | 0.225*** | 0.177*** | 0.010 | 0.000 | 0.017 | 0.010 | 0.225*** | 0.017 |
| Eh | | 0.024 | 0.026 | 0.020 | 0.024 | 0.050** | 0.037* | 0.044* | 0.030 | 0.026 | 0.050** |
| POM | | 0.172*** | 0.172*** | 0.164*** | 0.164*** | 0.020 | 0.020 | 0.017 | 0.017 | 0.172*** | 0.020 |
| TN | | 0.036 | 0.136*** | 0.014 | 0.122*** | 0.000 | 0.017 | 0.014 | 0.017 | 0.136*** | 0.017 |
| Richness | | 0.166*** | 0.071** | 0.153*** | 0.056* | 0.020 | 0.030 | 0.020 | 0.022 | 0.166*** | 0.030 |
| Abundance of core taxa | | 0.149*** | 0.281*** | 0.137*** | 0.248*** | 0.020 | 0.052** | 0.020 | 0.046** | 0.281*** | 0.052** |
|  | | Stochasticity ratio | | | | | | | | | |
| Depth | | 0.390*** | 0.217*** | 0.352*** | 0.196*** | 0.014 | 0.017 | 0.017 | 0.014 | 0.390*** | 0.017 |
| PDI | | 0.045 | 0.022 | 0.049* | 0.010 | 0.074*** | 0.066*** | 0.075*** | 0.071*** | 0.049* | 0.075*** |
| Tp | | 0.049* | 0.049* | 0.030 | 0.030 | 0.093*** | 0.058** | 0.095*** | 0.060** | 0.049* | 0.095*** |
| PWC | | 0.064* | 0.064* | 0.032 | 0.032 | 0.020 | 0.017 | 0.020 | 0.020 | 0.064* | 0.020 |
| pH | | 0.093*** | 0.073** | 0.097*** | 0.080** | 0.000 | 0.014 | 0.020 | 0.020 | 0.097*** | 0.020 |
| Eh | | 0.024 | 0.014 | 0.022 | 0.010 | 0.040* | 0.024 | 0.041* | 0.024 | 0.024 | 0.041* |
| POM | | 0.065* | 0.065* | 0.036 | 0.036 | 0.010 | 0.014 | 0.017 | 0.017 | 0.065* | 0.017 |
| TN | | 0.028 | 0.072** | 0.024 | 0.030 | 0.048** | 0.036* | 0.057** | 0.032 | 0.072** | 0.057** |
| Richness | | 0.163*** | 0.047 | 0.168*** | 0.025 | 0.017 | 0.046** | 0.014 | 0.037* | 0.168*** | 0.046** |
| Abundance of core taxa | | 0.150*** | 0.256*** | 0.138*** | 0.233*** | 0.020 | 0.048** | 0.020 | 0.047** | 0.256*** | 0.048** |

Correlations of bacterial composition and stochasticity ratio with environmental variables, richness, and relative abundance of core taxa in the active and permafrost layers. The correlations are analyzed by a modified Mantel test based on linear models. The relative importance of composition and assembly processes (Y) and each variable (X) are either log-transformed or not before fitting the models, to test linear (Y ~ X), logarithmic (Y ~ lnX), exponential (lnY ~ X), and power law (lnY ~ lnX) relationship. The best models are selected based on Mantel’ r. PDI, permafrost degradation index; Tp, permafrost temperature; PWC, permafrost water content; Eh, redox potential; POM, permafrost organic matter; TN, total nitrogen. Asterisks indicate statistical significance (****p* < 0.001, ***p* < 0.01, and **p* < 0.05).

**Table S5. Characteristics of nodes and edges in bacterial co-occurrence networks.**

|  | | Main-layer | | | | | | |
| --- | --- | --- | --- | --- | --- | --- | --- | --- |
|  | | | Active layer | | | Permafrost layer | | |
| No. of node | | | 960 | | | 458 | | |
| No. of edge | | | 21833 | | | 2123 | | |
| Proportion of negative edge (%) | | | 30.27 | | | 4.9 | | |
| Proportion of positive edge (%) | | | 69.73 | | | 95.1 | | |
|  | Sub-layer | | | | | | | |
|  | | | TAL | SAL | FFL | | TPL | SPL |
| No. of node | | | 795 | 980 | 991 | | 931 | 805 |
| No. of edge | | | 25818 | 25251 | 24918 | | 22520 | 12228 |
| Proportion of negative edge (%) | | | 39.18 | 43.81 | 39.33 | | 33.75 | 23.71 |
| Proportion of positive edge (%) | | | 60.82 | 56.19 | 60.67 | | 66.25 | 76.29 |
|  | Active layer | | | | | | | |
|  | | | S1 | S2 | S3 | | S4 | S5 |
| No. of node | | | 943 | 805 | 917 | | 855 | 862 |
| No. of edge | | | 25174 | 25505 | 24631 | | 20164 | 12499 |
| Proportion of negative edge (%) | | | 54.62 | 37.38 | 38.31 | | 40.74 | 35.26 |
| Proportion of positive edge (%) | | | 45.38 | 62.62 | 61.69 | | 59.26 | 64.74 |
|  | Permafrost layer | | | | | | | |
|  | | | S1 | S2 | S3 | | S4 | S5 |
| No. of node | | | 541 | 582 | 420 | | 510 | 415 |
| No. of edge | | | 6089 | 6873 | 3516 | | 6344 | 4074 |
| Proportion of negative edge (%) | | | 24.11 | 20.78 | 21.25 | | 30.77 | 10.68 |
| Proportion of positive edge (%) | | | 75.89 | 79.22 | 78.75 | | 69.23 | 89.32 |

Variations in numbers (NO.) of node and edge, as well as proportions of negative and positive edge across the main- and sub-layers, and the gradient of permafrost degradation. Sample sizes of sub-layers are n = 15, 31, 10, 49, and 20 for top-active layer (TAL), sub-active layer (SAL), frozen fringe layer (FFL), top-permafrost layer (TPL), and sub-permafrost layer (SPL), respectively. Sample sizes of sites in the active layer are n = 6, 7, 11, 12, and 15 for S1, S2, S3, S4, and S5, respectively. Sample sizes of sites in the permafrost layer are n = 19, 18, 14, 13, and 10 for S1, S2, S3, S4, and S5, respectively.

**Table S3. Composition of core taxa.**

**Table S6. Depth and grouped information of collected samples.**

**Table S7. Statistics of sample sequencing.**

**Table S8. Interaction of among major core and other taxa.**

**Table S9.** **Interaction of core taxa.**
